# Downregulation of Keap1 Confers Features of a Fasted Metabolic State

**DOI:** 10.1016/j.isci.2020.101638

**Published:** 2020-10-06

**Authors:** Elena V. Knatko, Michael H. Tatham, Ying Zhang, Cecilia Castro, Maureen Higgins, Sharadha Dayalan Naidu, Chiara Leonardi, Laureano de la Vega, Tadashi Honda, Julian L. Griffin, Ronald T. Hay, Albena T. Dinkova-Kostova

**Affiliations:** 1 Jacqui Wood Cancer Centre, Division of Cellular Medicine, School of Medicine, University of Dundee, Dundee, Scotland DD1 9SY, UK; 2 Centre for Gene Regulation and Expression, School of Life Sciences, University of Dundee, Dundee, DD1 5EH, Scotland, UK; 3 Department of Biochemistry and the Cambridge Systems Biology Centre, University of Cambridge, 80 Tennis Court Road, Cambridge, CB2 1QW, UK; 4 Department of Chemistry and Institute of Chemical Biology & Drug Discovery, Stony Brook University, Stony Brook, NY 11794-3400, USA; 5 Section of Biomolecular Medicine, Department of Metabolism, Digestion and Reproduction, Imperial College London, South Kensington, London SW7 2AZ, UK; 6 Department of Pharmacology and Molecular Sciences and Department of Medicine, Johns Hopkins University School of Medicine, Baltimore, MD 21205, USA

**Keywords:** Human Metabolism, Molecular Biology, Omics

## Abstract

Transcription factor nuclear factor erythroid 2 p45-related factor 2 (Nrf2) and its main negative regulator, Kelch-like ECH-associated protein 1 (Keap1), are at the interface between redox and intermediary metabolism, allowing adaptation and survival under conditions of oxidative, inflammatory, and metabolic stress. Nrf2 is the principal determinant of redox homeostasis, and contributes to mitochondrial function and integrity and cellular bioenergetics. Using proteomics and lipidomics, we show that genetic downregulation of Keap1 in mice, and the consequent Nrf2 activation to pharmacologically relevant levels, leads to upregulation of carboxylesterase 1 (Ces1) and acyl-CoA oxidase 2 (Acox2), decreases triglyceride levels, and alters the lipidome. This is accompanied by downregulation of hepatic ATP-citrate lyase (Acly) and decreased levels of acetyl-CoA, a trigger for autophagy. These findings suggest that downregulation of Keap1 confers features of a fasted metabolic state, which is an important consideration in the drug development of Keap1-targeting pharmacologic Nrf2 activators.

## Highlights

• Keap1 downregulation in mice increases Ces1 and Acox2 and decreases triglyceride levels
• Genetic interference with Keap1/Nrf2 alters the murine lipidome
• Reduced expression of Keap1 lowers hepatic levels of acetyl-CoA
• Deleting constitutively active Nrf2 decreases tubulin acetylation and autophagic flux

## Graphical Abstract

**Figure:**
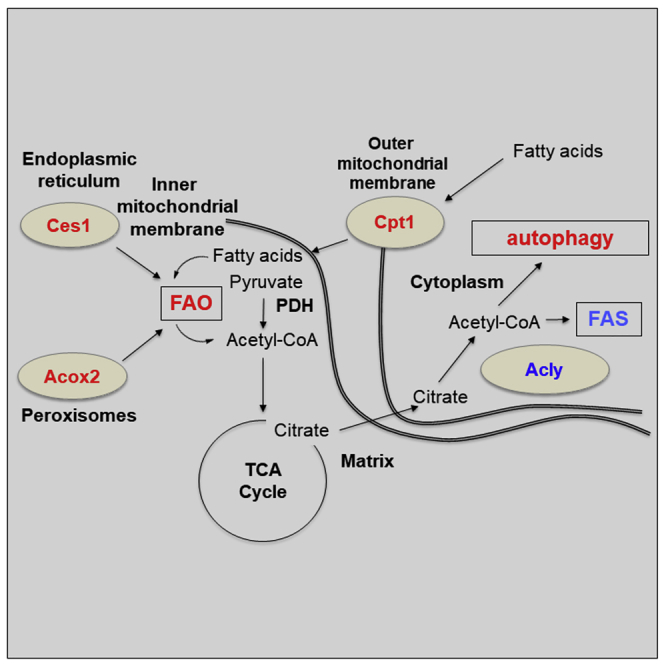

## Introduction

Kelch-like ECH-associated protein 1 (Keap1) is the mammalian sensor for electrophiles and oxidants and the main negative regulator of transcription factor nuclear factor erythroid 2 p45-related factor 2 (Nrf2, gene name *NFE2L2*). Together, Keap1 and Nrf2 form a tightly coupled sensor/transducer system that orchestrates the expression of a large network of genes encoding proteins, which are essential for adaptation and survival under conditions of oxidative, electrophilic, and inflammatory stress (Yamamoto et al., 2018). Genetic disruption of Nrf2 renders cells and animals much more sensitive to damage by electrophiles, oxidants, and inflammatory agents when compared with their wild-type counterparts; conversely, pharmacologic induction of Nrf2-dependent genes very effectively protects against electrophiles, oxidants, and pro-inflammatory agents in numerous animal models of chronic disease, and has health benefits in humans (Hayes and Dinkova-Kostova, 2014).

Under homeostatic conditions, Keap1 acts as a substrate adapter of a Cullin RING E3-ubiquitin Ligase (CRL), containing Cul3 and Rbx1, which continuously targets Nrf2 for ubiquitination and proteasomal degradation (Cullinan et al., 2004; Kobayashi et al., 2004; Zhang et al., 2004). In response to electrophiles and oxidants (termed inducers), which recognize and chemically modify specific cysteine residues of Keap1 (Dayalan Naidu and Dinkova-Kostova, 2020; Dinkova-Kostova et al., 2002), ubiquitination of Nrf2 is inhibited, leading to its stabilization and nuclear accumulation. Nuclear Nrf2 coordinately activates transcription of nearly 500 genes (Malhotra et al., 2010), the protein products of which are extraordinarily versatile and, by a range of mechanisms—including direct antioxidant activity, obligatory 2-electron reduction reactions, conjugation with endogenous ligands, recognition, repair and removal of damaged proteins—serve as critical cytoprotective defenses to eliminate a wide variety of potentially damaging agents and to restore redox balance.

In addition to genes encoding a large number of enzymes for drug metabolism, glutathione- and thioredoxin-related biosynthesis, and regeneration, in proliferating cells, such as those in the gastrointestinal epithelium, Nrf2 controls expression of malic enzyme 1 (ME1), isocitrate dehydrogenase 1 (IDH1), and the pentose phosphate pathway (PPP) enzymes glucose-6-phosphate dehydrogenase (G6PDH) and 6-phosphogluconate dehydrogenase (6PGD) (Mitsuishi et al., 2012; Thimmulappa et al., 2002; Wu et al., 2011); together, these four enzymes are principally responsible for NADPH generation. As NADPH is the main provider of reducing equivalents for redox and biosynthetic reactions, this critical function places Nrf2 at the interface between redox and intermediary metabolism. We previously reported that Nrf2 also affects cellular metabolism by improving mitochondrial function and bioenergetics (Holmstrom et al., 2013), in part by promoting fatty acid oxidation (FAO). In fact, FAO was enhanced in mouse embryonic fibroblast (MEF) cells and isolated mitochondria from Keap1-knockdown (Keap1-KD, with constitutive Nrf2 pathway activation due to downregulation of expression of Keap1) mice, whereas it was impaired in their Nrf2-knockout (Nrf2-KO) counterparts (Ludtmann et al., 2014). To gain further insights of the role of Nrf2 in lipid metabolism, in the current study, we used mitochondria-enriched preparations from the murine liver, an organ of high metabolic activity, and organoids from mouse small intestine, where the majority of the end absorption of nutrients takes place. In addition, some of our investigations included the murine colon, because high-fat diet accelerates progression of colorectal cancer in mice (Cai et al., 2015; Fu et al., 2019), and obesity, the prevalence of which is increasing worldwide (Bluher, 2019), is a colorectal cancer risk factor in humans (Kuipers et al., 2015).

Using proteomics and lipidomics, we demonstrate that downregulation of Keap1 in mice, and consequent Nrf2 activation to pharmacologically relevant levels, leads to induction of carboxylesterase 1 (Ces1) and acyl-CoA oxidase 2 (Acox2), enzymes involved in lipid catabolism; decreases triglyceride levels; and confers a distinct fatty acid profile. At the same time, hepatic ATP-citrate lyase (Acly) is suppressed and levels of its enzymatic product, acetyl coenzyme A (acetyl-CoA), are decreased, which is a trigger for autophagy. Together, these findings suggest that downregulation of Keap1 confers features of a fasted metabolic state. Understanding this is important, as dysregulation (either down- or upregulation) of Keap1/Nrf2 function is associated with disease risk in humans, including chronic obstructive pulmonary disease, cardiovascular and neurodegenerative diseases, as well as cancer (Cho et al., 2015; Quinti et al., 2017; Rojo de la Vega et al., 2018; von Otter et al., 2010). Furthermore, the Keap1/Nrf2 system is now considered a drug target, with a number of small molecule pharmacologic activators currently being in various stages of clinical development (Cuadrado et al., 2019).

## Results

### Genetic Interference with Keap1/Nrf2 Affects the Abundance of Metabolic Proteins

**Figure 1:**
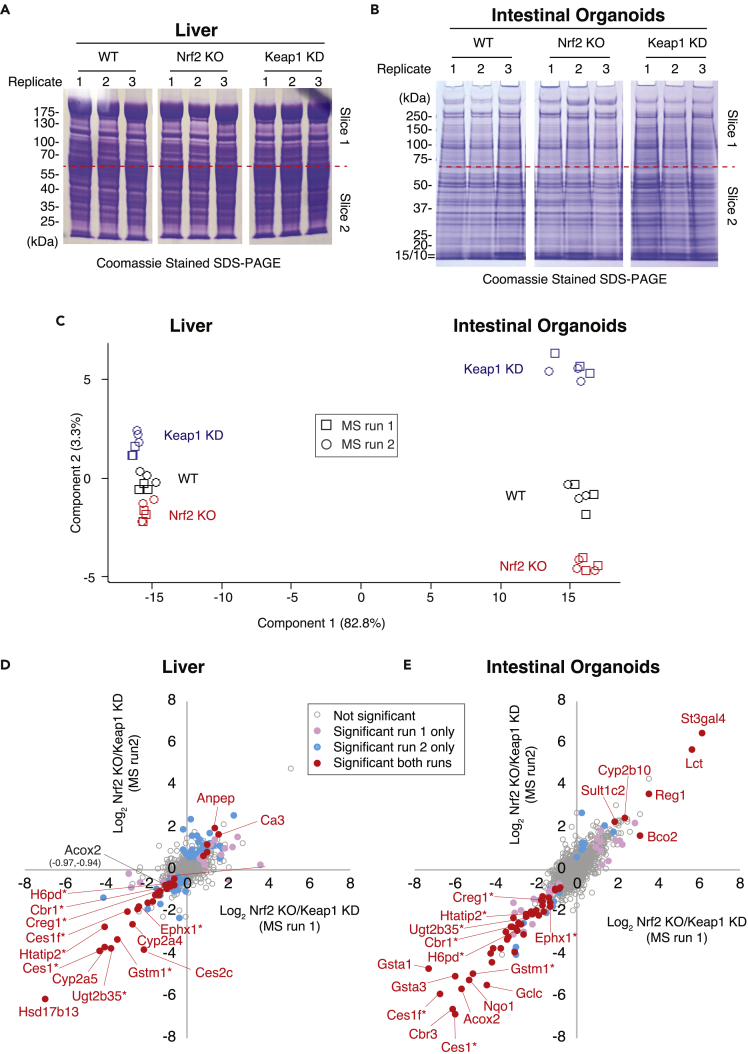
Proteomic Analyses of Mitochondria-Enriched Preparations from Liver and Early-Passage Intestinal Organoids from Wild-Type (WT), Nrf2-Knockout (Nrf2-KO), and Keap1-knockdown (Keap1-KD) Mice (A and B) Coomassie-stained SDS-PAGE gel of protein samples prepared from mitochondria-enriched fraction from liver (A) or intestinal organoids (B) from mice with the indicated genetic alterations. (C) Principal-component analysis (PCA) of 906 proteins with intensities reported in all samples in all MS runs in both experimental systems. Normalized LFQ intensities were log_2_ transformed and *Z*-scored by average log_2_ LFQ before PCA. (D and E) Summary of the quantitative data from two MS runs each for the enriched mitochondria samples from liver (D) and intestinal organoids (E). Proteins are colored by statistical criteria for outlier status (see text for statistical methods). Red entries are described further in Tables S1 and S2. The indicated proteins were found in all four MS runs to be significantly different between Nrf2-KO and Keap1-KD genotypes. Gene names: Cbr1: carbonyl reductase [NADPH] 1, Ces1: liver carboxylesterase 1, Ces1f: carboxylesterase 1F, Creg1: protein CREG1, Ephx1: epoxide hydrolase 1, Gstm1: glutathione S-transferase Mu 1, H6pd: GDH/6PGL endoplasmic bifunctional protein, Htatip2: oxidoreductase HTATIP2, Ugt2b35: UDP-glucuronosyltransferase. All data can be found in Data S1, Quantitative proteomics data. See also Figure S1.

To identify the protein components of metabolic pathways displaying altered expression in response to genetic interference with Keap1/Nrf2, a proteomic analysis was conducted. Mitochondria were enriched by differential centrifugation from (1) liver and (2) early-passage (p1) intestinal organoids prepared from wild-type (WT), Nrf2-KO, and Keap1-KD mice (Knatko et al., 2015; Taguchi et al., 2010). Proteins from mitochondria-enriched preparations, in triplicate, were separated by SDS-PAGE and visualized by Coomassie staining (Figures 1A and 1B). Tryptic peptides were extracted and analyzed by liquid chromatography-tandem mass spectrometry (LC-MS/MS) with two different run parameters using MaxQuant (Cox and Mann, 2008) for label-free Quantitation and Perseus (Tyanova et al., 2016) for bioinformatic analysis. Principal-component analysis (PCA) showed separation by tissue type (Figure 1C, component 1), as well as by genotype (Figure 1C, component 2), with the WT mouse samples spatially positioned between the two mutants (see Data S1, and Table S1 for details). This is a clear indication of the opposing effects of Nrf2 and Keap1 interference. Individual comparisons of Nrf2-KO and Keap1-KD with WT identified groups of proteins significantly up- or down-regulated by the mutations, however, to simplify further analysis, the ratio of Nrf2-KO/Keap1-KD was used, with any protein whose abundance is dependent on Nrf2 having a low Nrf2-KO/Keap1-KD ratio (or negative log_2_ ratio). Volcano plots of these comparisons for each MS run for liver (Figures S1A and S1B) and intestinal organoids (Figures S1C and S1D) indicated that the majority of quantified proteins do not change. A small group of proteins showed significant differences between Nrf2-KO and Keap1-KD genotypes (Figure 1D for liver and 1E for organoids), with nine defined as significantly altered (Student's t test, false discovery rate 0.1, S0 0.1) between the two genotypes in all four MS runs. These include enzymes involved in xenobiotic metabolism, namely, glutathione *S*-transferase μ1 (Gstm1), carbonyl reductase 1 (Cbr1), the endoplasmic reticulum (ER) enzymes epoxide hydrolase 1 (Ephx1), UDP-glucuronosyltransferase (Ugt2b35), liver carboxylesterase 1 (Ces1), carboxylesterase 1f (Ces1f), and hexose-6-phosphate dehydrogenase (H6pd), the initial enzyme of a PPP inside the ER that generates NADPH for ER enzymes. In addition, NADPH-binding short-chain oxidoreductase family member Htatip2 and endocytic-lysosomal compartment-residing protein Creg1 were also less abundant in the Nrf2-KO compared with the Keap1-KD genotype.

**Figure 2:**
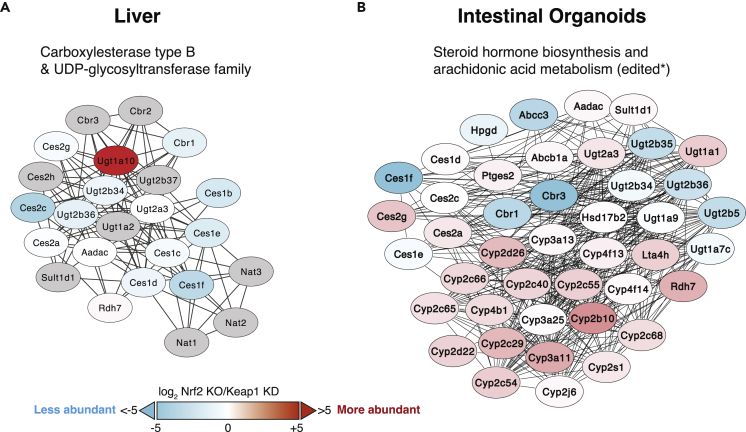
Clusters of Metabolic Proteins Identified by STRING Functional Group Enrichment Analyses (A and B) Network of proteins identified by STRING with both functional enrichments and quantitative relationships in mitochondria-enriched preparations from livers (A) and early-passage intestinal organoids (B) from Nrf2-knockout (Nrf2-KO) and Keap1-knockdown (Keap1-KD) mice. Networks created by STRING “Proteins with values/ranks” tool (Szklarczyk et al., 2019) were rendered in Cytoscape (Shannon et al., 2003) to overlay ratio values (colors). Gray proteins were not identified in the experiments but were included by STRING. For edges, a minimum interaction score of 0.4 (medium confidence) was applied, with disconnected nodes and subnetworks hidden. Some node positions have been adjusted in highly interacting regions to show names. This type of analysis identified clusters of metabolic proteins within the Ces1 and Ugt families in livers (A) and proteins within the Ces1 and Cyp families in intestinal organoids (B) as statistically significantly different between the Nrf2-KO and Keap1-KD genotypes. For other protein clusters, see Figures 3, S2, and S3.

**Figure 3:**
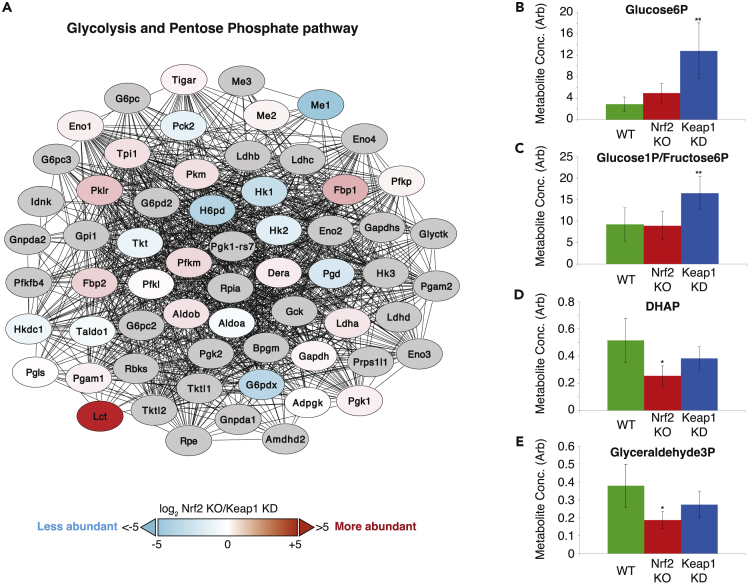
Genetic Interference with Keap1/Nrf2 Affects the Abundance of Glycolytic Enzymes and Metabolites (A–E) (A) Networks created by STRING “Proteins with values/ranks” tool (Szklarczyk et al., 2019) were rendered in Cytoscape (Shannon et al., 2003) to overlay ratio values (colors). Gray proteins were not identified in the experiments but were included by STRING. For edges, a minimum interaction score of 0.4 (medium confidence) was applied, with disconnected nodes and subnetworks hidden. Some node positions have been adjusted in highly interacting regions to show names. ∗Edited networks have had proteins not identified in the proteomics analysis removed for brevity. Full details of STRING data can be found in Data S2. In addition to clusters of proteins shown in Figures 2B and S3, this type of analysis identified clusters of proteins involved in glycolysis and the pentose phosphate pathway as statistically significantly different between the Nrf2-KO and Keap1-KD genotypes of organoid preparations. (B–E) Concentration of glucose-6-phosphate (B), glucose-1-phosphate/fructose-6-phosphate (C), dihydroxyacetone phosphate (DHAP) (D), and glyceraldehyde 3-phosphate (E) in colon tissue of C57BL/6 mice. Green bars represent wild-type (WT) mice, red bars Nrf2-knockout (Nrf2-KO) mice, and blue bars Keap1-knockdown (Keap1-KD) mice. ∗ 0.05 > p > 0.01; ∗∗ 0.01 > p > 0.001. See also Figure S4 and Table S3.

To extract more general biological patterns, STRING analysis was used to determine if any functionally related proteins showed similar changes between the two genotypes. Samples derived from liver provided fewer protein identifications and fewer functional enrichments than those from organoids. Liver network clusters with the highest enrichment score contained a number of carboxylesterases (Ces1 members) and UDP-glucuronosyltransferases (Ugt proteins), with lower abundances in Nrf2-KO (Figure 2A; see also Data S2, STRING functional group enrichment analysis, related to Figures 2 and 3, and Table S2). Curiously, an exception among members of the Ugt family of enzymes was Ugt1a10, the abundance of which was higher in Nrf2-KO than Keap1-KD. As transcription of Ugt1a10 is regulated by both Nrf2 and the aryl hydrocarbon receptor (AhR) (Kalthoff et al., 2010) and the two transcription factors engage in crosstalk (Hayes et al., 2009; Yeager et al., 2009), this finding suggests that binding of AhR to the promoter of Ugt1a10, and consequently its expression, might be enhanced in the absence of Nrf2. Few other networks showed coordinated changes in liver samples, although clusters of proteins with roles in protein processing in ER and signal peptidase complex (Figure S2A) and proteins involved in mitochondrial complex I biogenesis (Figure S2B) showed quantitatively modest, but statistically significant differences.

In organoids, due to the greater differences between the genotypes, it was possible to identify a higher number of significant network clusters. In close agreement with the liver data, organoid network clusters with the highest enrichment score also contained carboxylesterases (Ces1 members) and UDP-glucuronosyltransferases (Ugt proteins), with lower abundances in Nrf2-KO compared with Keap1-KD (Figure 2B). STRING also clustered in this network a group of cytochrome P450 (Cyp) proteins, which were more abundant in Nrf2-KO organoids. As AhR is a major transcriptional regulator of the Cyp family of enzymes (Androutsopoulos et al., 2009), this finding further supports the possibility that Nrf2 deficiency promotes AhR binding to its cognate promoter sequences. As expected, proteins involved in glutathione metabolism were much more abundant in Keap1-KD samples (Figure S3A). Moreover, proteins involved in glycolysis and the PPP were also significantly changed in expression (Figure 3A), although not in a coordinated fashion. Extracellular matrix proteins were much more abundant in organoid preparations from Nrf2-KO than Keap1-KD mice (Figure S3B). Finally, a group of DNA replication and repair proteins were modestly more abundant in Keap1-KD than Nrf2-KO (Figure S3C) organoids.

The effects of Nrf2 on the levels of enzymes involved in glycolysis were also apparent at the metabolic level. LC-MS of metabolites in colon tissue extracts showed dramatic changes in glycolysis, especially in Keap1-KD mice. Metabolic changes included glucose 6-phosphate (Figure 3C) and fructose 6-phosphate (Figure 3D), involved in the first steps of the pathway, which were significantly higher in colons of Keap1-KD mice compared with WT, whereas metabolites such as dihydroxyacetone phosphate (Figure 3E) and glyceraldehyde 3-phosphate (Figure 3F), involved in the later steps of glycolysis, were lower than in WT. These results are consistent with previously reported metabolic flux analyses using [1,2-^13^C_2_] glucose-containing medium in MEF cells, where glucose oxidation and entry of oxaloacetate and acetyl-CoA into the tricarboxylic acid (TCA) cycle were found to be significantly reduced in Nrf2-KO compared with WT cells, whereas Keap1-knockout cells showed a significant increase in substrate entry into the TCA cycle (Singh et al., 2013).

In addition to glycolysis and the PPP, gluconeogenesis also affects the levels of glucose 6-phosphate. Examination of our proteomics data for gluconeogenesis-related enzymes did not reveal any consistent differences among the genotypes, with the exception of hexokinase 1 (Hk1) and glycerol-3-phosphate dehydrogenase 2 (Gpd2), which were significantly differentially abundant in organoids; Hk1 was also approaching significance in liver samples (Table S3 and Figures S4A and S4B). These results are in agreement with the similar hepatic expression of the key gluconeogenic enzymes phosphoenolpyruvate carboxykinase 1 (Pepck) and glucose-6-phosphatase (G6pase) in WT and Keap1-KD mice fed standard diet, and comparable glucose production upon induction of gluconeogenesis in primary hepatocytes from these mice (Slocum et al., 2016). Notably, however, under conditions of high-fat-diet feeding, the expression of both Pepck and G6pase was ∼30% lower in Keap1-KD compared with WT mice, suggesting Nrf2-mediated repression of gluconeogenesis (Slocum et al., 2016).

### Nrf2 Regulates Gene Expression of Carboxylesterase 1 (Ces1) and Acyl-CoA Oxidase 2 (Acox2)

**Figure 4:**
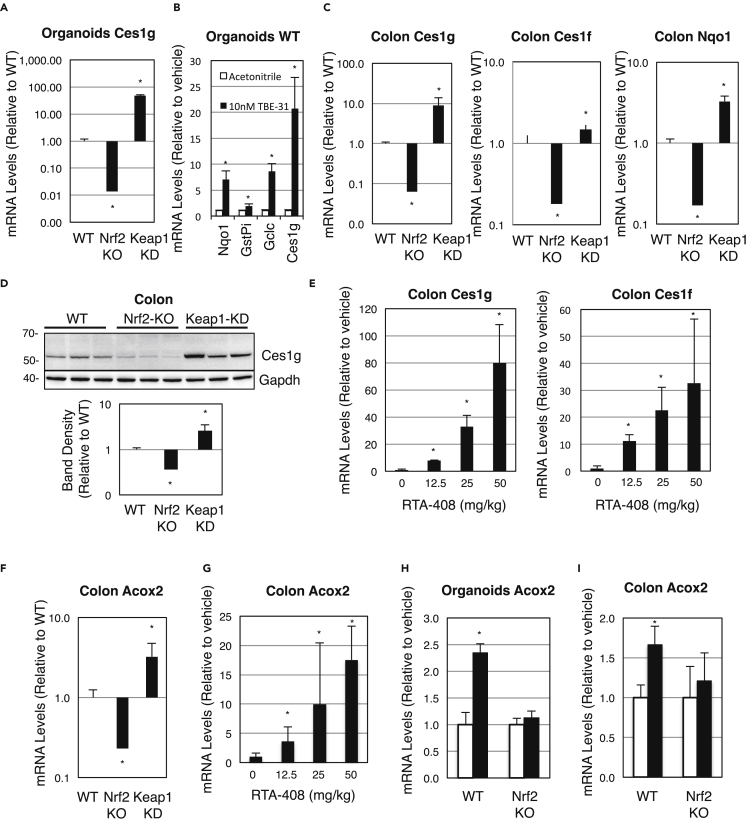
Ces1 and Acox2 Are Transcriptional Targets of Nrf2 (A) mRNA levels for Ces1g in cultures (n = 3) of intestinal organoids from wild-type (WT), Nrf2-knockout (Nrf2-KO), and Keap1-knockdown (Keap1-KD) C57BL/6 mice. (B) mRNA levels for Nqo1, Gstp, Gclc, and Ces1g in cultures (n = 3) of intestinal organoids from WT C57BL/6 mice that had been treated with vehicle (0.1% acetonitrile) or TBE-31 (10 nM) for 16 h. (C) mRNA levels for Ces1g, Ces1f, and Nqo1 in colon tissue of WT, Nrf2-KO, and Keap1-KD C57BL/6 mice (n = 3). (D) Protein levels for Ces1g in colon tissue of WT, Nrf2-KO, and Keap1-KD C57BL/6 mice (n = 3). (E) mRNA levels for Ces1g in colon tissue of male C57BL/6 WT mice (n = 3–4) that had been treated with vehicle (1% DMSO in corn oil) or RTA-408, *per os*, 3 times, 24 h apart; colon tissue was harvested 6 h after the last dose. (F) mRNA levels for Acox2 in colon tissue of WT, Nrf2-KO, and Keap1-KD C57BL/6 mice (n = 5). (G) mRNA levels for Acox2 in colon tissue of male WT C57BL/6 mice (n = 3–4) that had been treated with vehicle (1% DMSO in corn oil) or RTA-408, *per os*, 3 times, 24 h apart; colon tissue was harvested 6 h after the last dose. (H) mRNA levels for Acox2 in cultured intestinal organoids (n = 3) from WT and Nrf2-KO C57BL/6 mice that had been treated with vehicle (0.1% acetonitrile, white bars) or TBE-31 (10 nM, black bars) for 16 h. (I) mRNA levels for Acox2 in the colon of female WT and Nrf2-KO C57BL/6 mice (n = 4–5). The animals were treated with TBE-31 (5 nmol/g body weight, 3 times, at 24-h intervals, *per os,* black bars) or vehicle (1% DMSO in corn oil, white bars) and fasted for 4 h before tissue harvesting 24 h after the last dose. ∗p < 0.05. See also Figures S5, S6, S7, and S11.

As the proteomic analyses identified several members of the carboxylesterase 1 (Ces1) family as some of the most statistically significant differentially abundant proteins among the three genotypes in both experimental systems, further investigations were conducted to validate the link. First, mRNA levels for Ces1g were found to be 47-fold higher in Keap1-KD than in WT organoids and 98% lower in Nrf2-KO than in WT organoids (Figure 4A). Second, when intestinal organoids from the three genotypes of mice were treated with a tricyclic cyanoenone (TBE-31, Figure S5A), a compound that reacts with cysteine 151 in Keap1, thereby activating Nrf2 (Dayalan Naidu et al., 2018), the pattern of expression of Ces1g was similar to that of classical Nrf2-target genes, such as Nqo1, Gstp1, and Gclc in early-passage (p3) cultures. Thus, TBE-31 induced Ces1g to high levels in WT (Figure 4B), but not Nrf2-KO organoids (Figure S5B), and its induction was greatly diminished in their Keap1-KD counterparts (Figure S5C). Third, in colon tissue from mice of the three genotypes, mRNA levels for Ces1g and Ces1f were 9- and 1.5-fold, respectively, higher in colon tissue from Keap1-KD mice in comparison with their WT counterparts, whereas these levels were 94% and 80% lower in colons of Nrf2-KO mice, again with a pattern among the genotypes typical of classical Nrf2-target genes (Figure 4C). In addition to changes in mRNA levels, protein levels of Ces1g were similarly affected by genetic disruption or activation of Nrf2 in colon tissues (Figure 4D). Furthermore, the pentacyclic cyanoenone RTA-408 (Figure S5A), which, like TBE-31, reacts with cysteine 151 in Keap1 to activate Nrf2 (Shekh-Ahmad et al., 2018), induced expression of Ces1g and Ces1f dose dependently in colons of WT mice (Figure 4E), in a way similar to that of classical Nrf2-target genes Nqo1 (Figure S5D) and Gclc (Figure S5E).

As in mouse cells and tissues, treatment with TBE-31 or the naturally occurring Nrf2 activator sulforaphane (SFN) upregulated the expression of CES1 in the human hepatoma cell line HepG2, which has high basal levels of CES1 (Figure S6A), in a manner resembling that of NQO1 (Figure S6B) and GCLC (Figure S6C). Silencing of Nrf2 (by RNAi–for *NFE2L2*) (Figure S6D) reduced the expression of CES1 by 35%–40% in HepG2 cells growing in either glucose-containing or glucose-free medium, and abolished CES1 upregulation by TBE-31 (Figure S6E). The Nrf2 dependence of the expression of CES1 was further confirmed by using the human colorectal cancer cell line DLD1 and its Nrf2-KO and Nrf2-gain-of-function mutant isogenic lines that were generated using CRISPR/Cas9 genome editing (Torrente et al., 2017): compared with Nrf2-WT, the mRNA levels for CES1 were 8.4-fold higher in Nrf2-gain-of-function DLD1 cells, whereas these levels were 75% lower in their Nrf2-KO counterparts (Figure S6F). In Nrf2-WT DLD1 cells, exposure to TBE-31 caused a concentration-dependent Nrf2 stabilization (Figure S6G, immunoblot), and a corresponding CES1 upregulation, which was also observed following treatment with SFN (Figure S6G, bar graph), whereas CES1 upregulation by TBE-31 was greatly diminished in Nrf2-KO DLD1 cells (Figure S6H). Thus, using proteomic, genetic, and pharmacologic approaches, we validated that genes encoding Ces1 family members are transcriptional targets of Nrf2 in human cell lines, in mouse intestinal organoid cultures, and *in vivo* in the murine colon.

Proteomic analysis of mitochondria-enriched preparations isolated from intestinal organoids of WT, Nrf2-KO, and Keap1-KD mice identified another differentially abundant protein of relevance to FAO, namely, acyl-CoA oxidase 2 (Acox2) (Figure 1E), a peroxisomal enzyme that catalyzes oxidation of CoA esters of branched-chain fatty acids and bile acid intermediates (Vanhove et al., 1993). Similar to the classical Nrf2 target Nqo1, mRNA levels for Acox2 were 3-fold higher than WT in colons of Keap1-KD, whereas those levels were 80% lower in colons of Nrf2-KO mice (Figure 4F). Oral administration of Nrf2 activator RTA-408 dose dependently induced gene expression of Acox2 in colons of WT animals (Figure 4G), similarly to Ces1g, Ces1f (Figure 4E), Nqo1 (Figure S5D), and Gclc (Figure S5E). In addition, Nrf2 activator TBE-31 induced expression of Acox2 in intestinal organoids from WT, but not Nrf2-KO mice (Figure 4H), in a manner similar to that of Nqo1, Gstp, Gclc, and Ces1g (Figures 4B and S5B). Both Acox2 (Figure 4I) and Nqo1 (Figure S5F) were induced by oral administration of TBE-31 in colons of WT, but not Nrf2-KO mice. Thus, as with Ces1, using both genetic and pharmacologic approaches, we validated that Acox2 is a transcriptional target of Nrf2 in intestinal organoid cultures and *in vivo* in the murine colon.

The levels of ACOX2 have been shown to be downregulated upon knockdown of Nrf2 (by RNAi for *NFE2L2*) in 293T human embryonic fibroblasts (Pang et al., 2014), suggesting that like in mice, human ACOX2 is a transcriptional target of Nrf2. Surprisingly, we found no evidence for Nrf2 dependence of ACOX2 regulation in several human cell lines (i.e., HepG2 [hepatoma], Caco2 [colorectal cancer], and IMR90 [normal lung fibroblasts]) that we tested, and its expression levels were below the limit of detection in DLD1 (colorectal cancer), A549 (lung cancer), and U2OS (osteosarcoma) cells, indicating cell type and/or species specificity. Thus, ACOX2 expression was not upregulated by treatment with TBE-31 or SFN in HepG2 (Figure S7A), Caco2 (Figure S7B), or IMR90 (Figure S7K) cells. A time course analysis in Caco2 cells showed that the basal mRNA levels for ACOX2 increased ∼2-fold at the 48-h time point, but there was no difference between vehicle- and SFN-treated cells (Figure S7E). Furthermore, knockdown of Nrf2 (by RNAi for *NFE2L2*) (Figure S7H) in Caco2 cells did not affect the mRNA levels for ACOX2 (Figure S7J). By contrast, the levels of the Nrf2-targets NQO1 (Figures S6B, S7C, S7F, and S7L) and AKR1B10 (Figures S7D, S7G, and S7M) were upregulated by the inducer and downregulated by the small interfering RNA (Figure S7I) treatments, as expected. Thus, it is unlikely that Nrf2 plays a role in the transcriptional regulation of ACOX2 in humans, indicating species differences.

### Downregulation of Keap1 Decreases the Levels of Triglycerides and Alters the Lipidome

**Figure 5:**
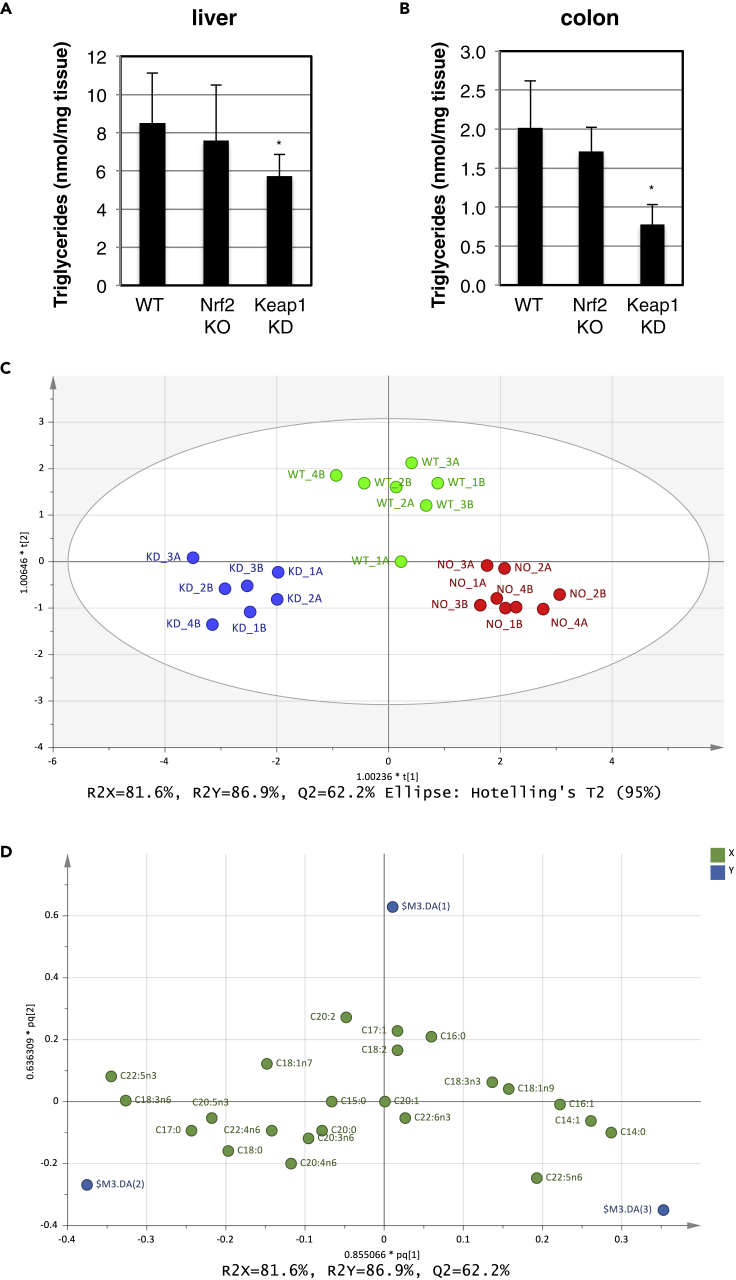
Nrf2 Alters the Lipidome (A and B) Levels of triglycerides in liver (A) and colon (B) of wild-type (WT), Nrf2-knockout (Nrf2-KO), and Keap1-knockdown (Keap1-KD) C57BL/6 mice (n = 4). ∗p < 0.05. (C) Orthogonal projection to latent structure-discriminant analysis (OPLS-DA) score plot from GC-MS data for total fatty acids. Seven independent biological replicates of colon tissue from WT (green), Keap1-KD (blue), and Nrf2-KO (red) C57BL/6 mice were included. (D) Plot showing the individual fatty acids driving the separation among the genotypes (loadings). The variables (i.e., fatty acids) are represented as green circles, while the direction of the discriminate variable from the classification matrix for each genotype (WT $M1-DA(1), Keap1-KD -$M1-DA(2), and Nrf2-KO $M1-DA(3)) is in blue.

In agreement with their function in lipid metabolism and our finding that genes encoding Ces1 and Acox2 are transcriptionally activated by Nrf2, levels of triglycerides were lower in livers (Figure 5A) and colons (Figure 5B) of Keap1-KD compared with WT mice. This is fully consistent with the lower hepatic triglyceride levels in Keap1-KD compared with WT mice that had been fed either regular chow or high-fat diet (Slocum et al., 2016). Notably, however, triglyceride levels in the corresponding tissues of Nrf2-KO mice were not significantly different from either WT or Keap1-KD animals (Figures 5A and 5B), indicating that Ces1 and Acox2 are not the only enzymes responsible for differences in triglycerides among the genotypes.

To better understand how fatty acid metabolism was affected by genotype, as suggested by differential expression of Ces1- and Acox2-encoding genes, fatty acids were esterified, after hydrolysis from lipids, and analyzed using gas chromatography-MS. Distinct fatty acid profiles were apparent for each genotype, with the WT again having a mid-point position between the other two genotypes (Figure 5C). Saturated and mono-unsaturated fatty acids, including C14:0, C14:1n9, and C16:0 were higher in Nrf2-KO mice, whereas polyunsaturated fatty acids, such as C22:5n3, C18:3n6, and C20:5n3, and the odd-chain fatty acid C17:0 were lower in Nrf2-KO mice (Figure 5D).

### Reduced Expression of Keap1 Lowers Hepatic Levels of Acetyl-CoA

**Figure 6:**
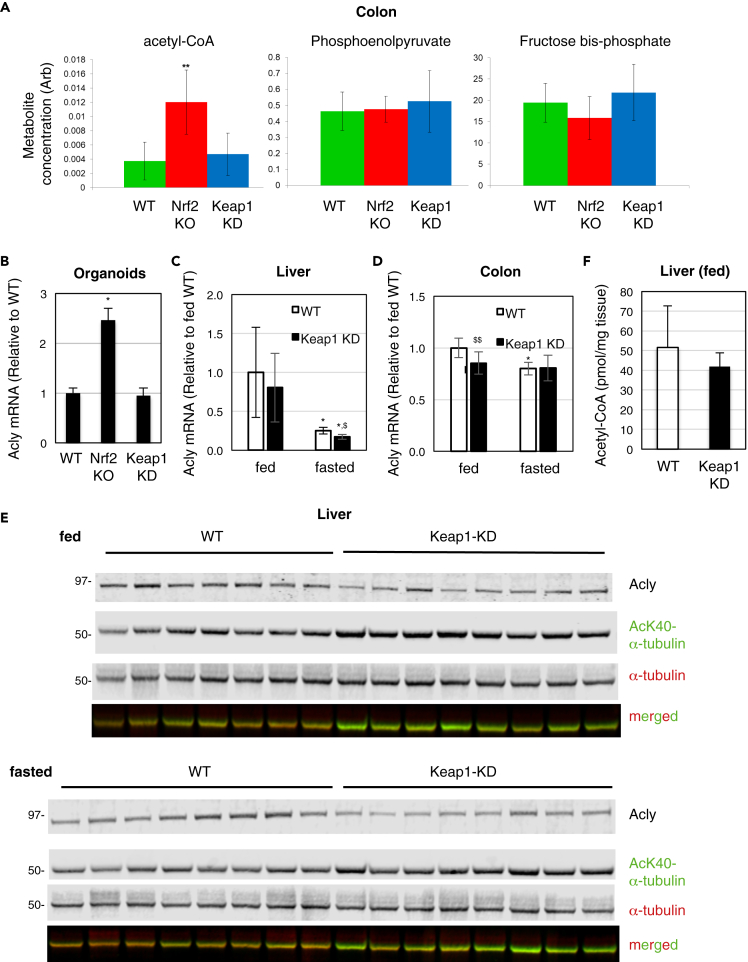
Downregulation of Keap1 Decreases the Hepatic Levels of Acetyl-CoA at Fed State and Increases the Acetylation of α-tubulin Following Fasting (A) Concentration of phosphoenolpyruvate, fructose bis-phosphate, and acetyl-CoA in colons of wild-type (WT, green bars), Nrf2-knockout (Nrf2-KO, red bars), and Keap1-knockdown (Keap1-KD, blue bars) C57BL/6 mice. ∗∗ 0.01 > p > 0.001. (B) mRNA levels for Acly in organoids from wild-type (WT), Nrf2-knockout (Nrf2-KO), and Keap1-knockdown (Keap1-KD) C57BL/6 mice. ∗p < 0.01. (C and D) mRNA levels for Acly in livers (C) and colons (D) of wild-type (WT) and Keap1-knockdown (Keap1-KD) female C57BL/6 mice (n = 8) that were either fed *ad libitum* or fasted for 18 h; *18S* used as a reference gene; ∗p < 0.01, in relation to fed state in respective genotype; ^$^p < 0.01 and ^$$^0.01 < p < 0.05, relative to respective WT. (E) Protein levels for Acly, AcK40-α-tubulin, and α-tubulin in livers from wild-type (WT) and Keap1-knockdown (Keap1-KD) female C57BL/6 mice (n = 7–8) that were either fed *ad libitum* or fasted for 18 h. (F) Levels of acetyl-CoA in livers of fed wild-type (WT) and Keap1-knockdown (Keap1-KD) female C57BL/6 mice (n = 8). See also Figures S8–S12.

Metabolomic analysis of colon tissue extracts showed that levels of acetyl-CoA were significantly higher in Nrf2-KO mice than in WT or Keap1-KD animals, whereas levels of phosphoenolpyruvate and fructose bis-phosphate did not differ among the genotypes (Figure 6A). The enzyme primarily responsible for the synthesis of cytosolic acetyl-CoA is ATP-citrate lyase (Acly). Correlating with differences in acetyl-CoA levels, mRNA levels for Acly were higher in organoid preparations from Nrf2-KO mice compared with their WT or Keap1-KD counterparts (Figure 6B). These differences between WT and Nrf2-KO genotypes are consistent with previous gene expression profiling and proteomics studies demonstrating that Nrf2 negatively regulates the gene expression of Acly in murine liver (Kitteringham et al., 2010; Yates et al., 2009). However, differences in abundance of Acly among the three genotypes were not apparent in our proteomics analysis. To address the possibility that high inter-individual variability may have masked differences in Acly expression among the genotypes, we first examined mRNA levels for Acly in the liver of *ad libitum*-fed WT and Keap1-KD mice. Based on the greater physiological relevance of Keap1 downregulation as opposed to Nrf2 absence, we focused our comparisons on the Keap1-KD and WT genotypes. Indeed, hepatic Acly mRNA levels of *ad libitum*-fed WT mice were highly variable among individual animals (n = 8), although these levels appeared lower in Keap1-KD compared with WT animals (Figures 6C and S8A). We hypothesized that the high inter-individual variability might be due to differences in feeding times, fasted the animals overnight, and found a dramatically decreased (by ∼80%), but much more uniform, hepatic Acly expression (Figure 6C). Levels of Acly mRNA in colon were similar in fed Keap1-KD and WT mice (Figures 6D and S8B), and compared with liver, these levels decreased much more modestly (by ∼20%) following fasting in WT mice, and did not change in KD animals (Figure 6D). Thus, high inter-individual variability and tissue specificity in Acly expression in fed animals provides one explanation for lack of statistically significant differences among the genotypes in our proteomics analysis.

Immunoblotting analysis of hepatic protein levels of Acly in individual mice further revealed that overall, Acly levels were lower in Keap1-KD compared with WT mice at both fed and fasted states (Figure 6E). Furthermore, levels of the Acly reaction product, acetyl-CoA, were lower in livers of *ad libitum*-fed Keap1-KD mice than in WT animals (Figures 6F and S8C). The levels of acetyl-CoA are also affected by the activity of acyl-CoA synthetase short-chain family member 2 (Acss2), which catalyzes the synthesis of acetyl-CoA from acetate (Berg, 1956). Similar to Acly, fasting caused a drop (by ∼80%) in the mRNA levels for Acss2, but in contrast to the lower expression of Acly in livers of fasted Keap1-KD compared with WT mice (Figure 6C), there was no difference in expression of Acss2 between genotypes at either fed or fasted state (Figure S8D). Curiously, the decrease in hepatic protein levels of Acly in fasted mice seemed to correlate with the order of tissue harvest/time after food withdrawal (Figure S8E). As expected, overnight fasting induced expression of a number of classical FAO enzymes, i.e., Cpt1, Cd36, Acads, Acadm, Acadl, and Acadvl, but interestingly, the extent of upregulation was blunted in livers of Keap1-KD animals compared with their WT counterparts (Figure S9A). By contrast, the levels of most enzymes involved in fatty acid synthesis (FAS), i.e., Acaca, Fasn, Scd1, Scd2, Elovl1, and Elovl6, were downregulated by fasting with no difference between genotypes (Figure S9B). In colon, fasting-mediated changes in expression of the classical enzymes involved in FAO and FAS were modest and similar between WT and Keap1-KD animals (Figures S10A and S10B). Notably, the levels of Ces1g and Acox2 were higher in livers and colons of Keap1-KD than WT mice at both fed and fasted states (Figures S11A and S11B).

Collectively, these results show that changes in Acly expression consequent to genetic interference with Keap1/Nrf2 are tissue specific as well as dependent on the nutrient intake (fed versus fasted) state of the animals. In a fed state, acetyl-CoA is directed out of mitochondria to the cytoplasm for use in FAS, whereas under fasted state, acetyl-CoA is channeled into mitochondria for ATP synthesis (Shi and Tu, 2015). Thus, the finding that hepatic levels of acetyl-CoA are lower in fed Keap1-KD compared with WT mice, together with our earlier observations of increased FAO upon Nrf2 activation (Ludtmann et al., 2014) suggest that Nrf2 activation by Keap1 downregulation under fed conditions has features of a fasted metabolic state.

**Figure 7:**
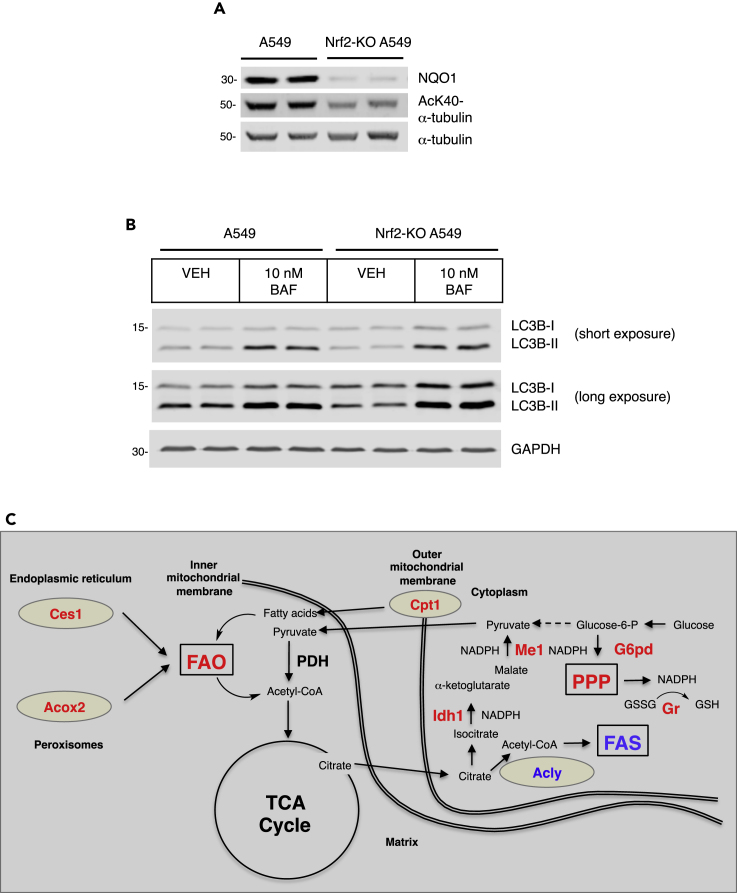
Deletion of Nrf2 in the Context of Mutant Keap1, Which Does Not Suppress Nrf2, Decreases the Acetylation of α-tubulin and Autophagic Flux (A) Levels of NQO1, AcK40-α-tubulin, and α-tubulin in whole-cell lysates of A549 and Nrf2-KO A549 cells. (B) Levels of LC3B-I (non-lipidated form) and LC3B-II (lipidated form) in whole-cell lysates of A549 and Nrf2-KO A549 cells that had been treated with vehicle (0.1% DMSO, VEH) or 10 nM bafilomycin A1 (BAF) for 16 h. GAPDH served as a loading control. See also Figure S12. (C) Downregulation of Keap1 has features of a fasted metabolic state. Nrf2 channels glucose through the pentose phosphate pathway by upregulating glucose-6-phosphate dehydrogenase (G6pd) and the enzymes of the pentose phosphate pathway (PPP) and enhances fatty acid oxidation (FAO) in part by upregulating Ces1 and Acox2, as well as the fatty acid transporter Cpt1, while inhibiting fatty acid synthesis (FAS) by downregulating Acly, and thus decreasing the levels of cytosolic acetyl-CoA. These features of a fasted metabolic state channel acetyl-CoA into the mitochondria for ATP synthesis and increase autophagic flux. The upregulation of the PPP, isocitrate dehydrogenase-1 (Idh1), and malic enzyme-1 (Me1) provides reducing equivalents (NADPH) for redox reactions and regeneration of reduced glutathione (GSH), which is catalyzed by the Nrf2-target enzyme glutathione reductase (Gr).

A decrease in acetyl-CoA levels is a trigger for autophagy (Marino et al., 2014). During autophagy, the levels of acetylated (AcK40)-α-tubulin increase, and this event is an essential requirement for starvation-induced autophagy (Geeraert et al., 2010; Marino et al., 2014). We found that levels of acetylated (AcK40)-α-tubulin were higher in hepatic tissue of both *ad libitum*-fed and fasted Keap1-KD mice than in their WT counterparts (Figures 6E and S8F). Consistent with Nrf2 activation promoting α-tubulin acetylation, compared with WT, the levels of (AcK40)-α-tubulin were higher in primary embryonic fibroblast (MEF) cells isolated from Keap1-KD mice, and lower in their Nrf2-KO counterparts (Figure S12). In close agreement with the mouse data, the levels of (AcK40)-α-tubulin were also higher in human lung cancer A549 cells when compared with CRISPR/Cas9-generated Nrf2-KO A549 cells (Figure 7A). The A549 cell line has constitutively high Nrf2 levels due to a homozygous mutation (G333C) in the Kelch domain of Keap1, the site of interaction between Keap1 and Nrf2 (Singh et al., 2006). As this experimental system (i.e., Nrf2-KO A549 and A549 cells) represents the two extreme conditions, namely, Nrf2 absence versus Nrf2 constitutive activation, we used it to examine autophagic flux. We found that, compared with Nrf2-KO A549 cells, autophagic flux was enhanced in the parental A549 cells, as evident by the higher levels of the lipidated form of the autophagosomal marker microtubule-associated protein 1A/1B light chain 3B (LC3B), (LC3B-II), and its further accumulation upon treatment with the autophagy inhibitor bafilomycin A1 (Figure 7B). This result is in agreement with the known involvement of Nrf2 in regulation of multiple genes that participate in macropautophagy and chaperone-mediated autophagy (Pajares et al., 2016, 2018).

## Discussion

The results from this study are in agreement with previous reports showing (1) dysregulated expression of lipid-metabolizing enzymes, including lower levels of Ces1g in livers of Nrf2-KO compared with WT mice (Kitteringham et al., 2010; Tanaka et al., 2012); (2) reduced hepatic expression of genes involved in FAS and desaturation in mice with high Nrf2 levels (Sharma et al., 2018; Wu et al., 2011; Yates et al., 2009); (3) increased high-fat-diet-induced levels of lipogenic enzymes in livers of Nrf2-KO compared with WT mice (Meakin et al., 2014); (4) lower ethanol-induced accumulation of free fatty acids in livers of hepatocyte-specific Keap1-knockout mice (Wu et al., 2012); (5) requirement for Nrf2 for hepatic Ces1g induction by the Nrf2 activator oltipraz (Zhang et al., 2012); and (6) higher mRNA levels for Ces1g and Ces1h in lungs of Keap1-knockout compared with WT mice (Paek et al., 2012). Taken together with knowledge that Nrf2 activation in proliferating cells, such as cultured MEF cells, as well as cells in the murine intestinal and forestomach epithelium, channels glucose through the PPP by upregulating glucose-6-phosphate dehydrogenase (G6pd) and enzymes of the PPP (Mitsuishi et al., 2012), our findings suggest that Nrf2 activation confers features of a fasted metabolic state (Figure 7C). Nrf2 activation enhances FAO in part by upregulating Ces1 and Acox2, as well as the uptake of fatty acids through Cd36, while inhibiting FAS by downregulating Acly, and thus decreasing the levels of acetyl-CoA. Notably, however, although Nrf2 activation does not equate typical fasting, the blunted response to fasting of the Keap1-KD mice suggests that Nrf2 activation provides quantitatively modest, but widespread preconditioning to fasting, allowing adaptation to the associated metabolic stress.

Ces1 enzymes catalyze the trans-esterification and hydrolysis of ester, thioester, or amide bonds within various substrates, including acyl glycerols to give free fatty acids and participate in fatty acid and cholesterol ester metabolism (Hosokawa et al., 2007), channeling fatty acids toward oxidation and away from storage (Ko et al., 2009). These enzymes are localized in the ER, which is physically connected with mitochondria (Rowland and Voeltz, 2012). Furthermore, physical contacts between mitochondria and ER lead to formation of specialized structures termed mitochondria-associated membranes, where critical metabolic processes, such as lipid trafficking, reactive oxygen species, and Ca^2+^ signaling occur, thereby allowing localized inter-organellar communication (Csordas et al., 2018; Scorrano et al., 2019). Acting in concert with uridine 5′-diphospho-glucuronosyltransferaseares (UGTs), Ces1 enzymes are also involved in xenobiotic metabolism, including the metabolism of cocaine and heroin and detoxification of organophosphate chemical weapons, such as sarin, soman, and tabun (Bencharit et al., 2003). The UGTs are drug-metabolizing enzymes encoded by classical Nrf2-target genes; indeed, we observed members of this family to be differentially expressed among the genotypes in the proteomics screen (Tables S1 and S2, and Figure 2), confirming the presence of ER proteins in our mitochondria-enriched fractions. Ces1g-knockout mice have reduced energy expenditure, increased lipogenesis, and postprandial hyperlipidemia due to increased secretion of chylomicrons, whereas Ces1g overexpression leads to increased FAO and reduced hepatic triglyceride levels (Quiroga et al., 2012; Xu et al., 2014). Most recently, it was shown that hepatocyte-specific overexpression of human CES1 in mice promotes FAO and attenuates high-fat/high-cholesterol/high-fructose diet- or alcohol-induced hepatic steatosis, inflammation, fibrosis, and hyperlipidemia, strongly suggesting a protective role of hepatic CES1 against metabolic disorders (Xu et al., 2020). Taken together with our findings of the regulation of CES1 by Nrf2, it can be concluded that one mechanism by which Nrf2 activation protects against metabolic disorders is through induction of CES1.

Although other transcription factors are also involved in regulating Ces1 expression, some similarities between Ces1g-KO and Nrf2-KO mice are noteworthy. Thus, Ces1 deficiency results in hepatosteatosis (Quiroga et al., 2012), and albeit to a much lower degree, there is evidence for microvesicular hepatic steatosis in Nrf2-KO animals (Chowdhry et al., 2010; Meakin et al., 2014; Sugimoto et al., 2010). Ces1g-KO mice are protected against development of atherosclerosis (Xu et al., 2017), as are Nrf2-KO mice (Polonen et al., 2019; Sussan et al., 2008). In addition, knockout of Ces1g decreases levels of cholesterol in plasma (Xu et al., 2017), as does Nrf2 deficiency (Meakin et al., 2014; Polonen et al., 2019), whereas plasma low-density lipoprotein levels are increased following chronic pharmacologic activation of Nrf2 by TBE-31 (Kostov et al., 2015). Taken together, these findings suggest that one mechanism by which Nrf2 activation affects lipid metabolism involves Ces1.

The beneficial effects of intermittent fasting have been consistently observed in numerous preclinical models of chronic disease, including obesity, diabetes, and neurodegenerative diseases, and although the clinical evidence is much more limited, benefits have been also noted in patients with metabolic disorders, such as obesity and insulin resistance (de Cabo and Mattson, 2019). A recent study in mice has shown that although the improvements in physical performance resulting from caloric restriction do not require Nrf2, the alterations in metabolic and protein homeostasis were Nrf2 dependent (Pomatto et al., 2020). Parallels can be drawn between reduced expression of Keap1/activation of Nrf2 and intermittent fasting. Similar to intermittent fasting, Nrf2 activation triggers adaptive responses resulting in improved glucose regulation, increased resistance to stress, and resolution of inflammation. Like Nrf2 activation, which counteracts and provides long-lasting protection against subsequent challenges (Gao et al., 2001), intermittent fasting leads to adaptive responses of long duration, which confer resistance to subsequent potentially damaging exposures and has inspired the search for targeted pharmacologic approaches that mimic the effects of fasting (de Cabo and Mattson, 2019). Our findings suggest that pharmacologic inhibition of Keap1 may offer such approach, particularly for conditions such as obesity-induced metabolic syndrome. Indeed, the anti-inflammatory, anti-lipogenic, and anti-fibrotic effects of Nrf2 pathway activation are particularly pronounced when mice are fed high-fat or high-fat plus high-fructose diet (Meakin et al., 2014; Sharma et al., 2018; Slocum et al., 2016). This notion is further supported by results from a human study showing that a 12-week intervention with the classical Nrf2 activator SFN (administered as broccoli sprout extracts) improved glucose control in obese patients with type 2 diabetes, as evidenced by the decrease in glycated hemoglobin and fasting blood glucose, which correlated with serum SFN concentration (Axelsson et al., 2017). Like TBE-31 and RTA-408, SFN inactivates Keap1 by reacting with cysteine 151 (Zhang and Hannink, 2003).

It should be pointed out that the level of Nrf2 activation in the Keap1-KD animals that we used in this study is relatively modest and comparable to levels observed following interventions with pharmacologic Nrf2 activators in both mice (Knatko et al., 2015) and humans (Dinkova-Kostova et al., 2007; Liu et al., 2020). This was an important consideration, particularly because Nrf2 activators, such as SFN and the pentacyclic cyanoenones bardoxolone methyl and RTA-408 (used in this study), are currently in clinical trials for multiple indications, including chronic kidney disease, liver disease, pulmonary arterial hypertension, mitochondrial myopathy, and autism spectrum disorder (Cuadrado et al., 2019). In addition, development of non-electrophilic compounds, which target the Nrf2-binding domain of Keap1 and consequently disrupt its protein-protein interactions with Nrf2, is also actively being pursued and has been the focus of a recent extensive virtual screen drug discovery effort, in which more than 1 billion compounds were assessed (Gorgulla et al., 2020).

### Limitations of the Study

The comparisons among WT, Nrf2-KO, and Keap1-KD genotypes provide confidence that most functional outcomes observed in this study are Nrf2 dependent. Nonetheless, we cannot exclude the possibility that some may be partially Nrf2 dependent. This is because, in addition to activating Nrf2, the lower expression of Keap1 in Keap1-KD animals is expected to affect the behavior of other (known as well as yet to be discovered) Keap1-binding partners, and the potential consequences have not been examined. Another limitation of this study is that, for most experiments, we have used female mice, and although highly unlikely, we cannot exclude the possibility that some of the responses of male mice could be different. Finally, future work is needed to elucidate the mechanisms by which Nrf2 acts as a negative regulator of the proteins identified in our proteomics screen.

### Resource Availability

#### Lead Contact

Albena T. Dinkova-Kostova (a.dinkovakostova@dundee.ac.uk).

#### Materials Availability

Materials are available from the corresponding author on request.

#### Data and Code Availability

All data are available in the main text or in Supplemental information and files. The mass spectrometry proteomics data have been deposited to the ProteomeXchange Consortium via the PRIDE partner repository with the dataset identifier PXD021639.

## Methods

All methods can be found in the accompanying Transparent Methods supplemental file.

## Supplemental Information

Document S1. Transparent Methods, Figures S1–S12, and Tables S1–S3

Data S1. Quantitative Proteomics Data, Related to Figure 1

Data S2. STRING Functional Group Enrichment Analysis, Related to Figures 2 and 3
