# Supplementary material for: Downregulation of Keap1 Confers Features of a Fasted Metabolic State
Source: iScience. 2020 Oct 6;23(10):101638. doi: 10.1016/j.isci.2020.101638 (PMC7575887; doi:10.1016/j.isci.2020.101638)
Supplement: Document S1. Transparent Methods, Figures S1–S12, and Tables S1–S3 [file mmc1.pdf]

iScience, Volume *23*

Supplemental Information

Downregulation of Keap1 Confers Features of a Fasted Metabolic State

Elena V. Knatko, Michael H. Tatham, Ying Zhang, Cecilia Castro, Maureen Higgins, Sharadha Dayalan Naidu, Chiara Leonardi, Laureano de la Vega, Tadashi Honda, Julian L. Griffin, Ronald T. Hay, and Albena T. Dinkova-Kostova

Liver

Uniprot Accession	Uniprot Locus	Protein name	Gene name	Average log_2 Nrf2 KO/Keap1 KD	Average $-log_{10}$ p-value Nrf2 KO v Keap1 KD	T-test Significant Nrf2 KO v Keap1 KD Run 1	T-test Significant Nrf2 KO v Keap1 KD Run 2	log_2 Nrf2/Keap1 Run 1	log_2 Nrf2/Keap1 Run 2	-log p-value Nrf2 KO v Keap1 KD Run 1	-log p-value Nrf2 KO v Keap1 KD Run 2
P48410	ABCD1_MOUSE	ATP-binding cassette sub-family D member 1	Abcd1	-1.79	2.22	+	+	-1.92	-1.66	2.61	1.83
P97449	AMPN_MOUSE	Aminopeptidase N	Anpep	1.64	2.05	+	+	1.38	1.90	2.60	1.51
P16015	CAH3_MOUSE	Carbonic anhydrase 3	Ca3	1.62	2.02	+	+	1.61	1.62	2.12	1.92
P48758	CBR1_MOUSE	Carbonyl reductase [NADPH] 1	Cbr1*	-0.97	2.57	+	+	-0.95	-0.98	2.90	2.25
Q8VCC2	EST1_MOUSE	Liver carboxylesterase 1	Ces1*	-4.09	1.97	+	+	-4.25	-3.92	1.73	2.21
Q8VCT4	CES1D_MOUSE	Carboxylesterase 1D	Ces1d	-0.68	2.63	+	+	-0.58	-0.78	2.73	2.54
H3BL34	H3BL34_MOUSE	Carboxylic ester hydrolase	Ces1e	-1.28	2.01	+	+	-1.41	-1.14	2.04	1.98
Q91WU0	CES1F_MOUSE	Carboxylesterase 1F	Ces1f*	-2.48	3.54	+	+	-2.89	-2.07	4.56	2.51
Q91WG0	EST2C_MOUSE	Acylcarnitine hydrolase	Ces2c	-2.98	1.88	+	+	-2.11	-3.85	1.85	1.90
Q8BJ64	CHDH_MOUSE	Choline dehydrogenase, mitochondrial	Chdh	-0.74	2.67	+	+	-0.76	-0.72	2.76	2.58
O88668	CREG1_MOUSE	Protein CREG1	Creg1*	-1.32	2.75	+	+	-1.36	-1.29	2.50	2.99
P15392	CP2A4_MOUSE	Cytochrome P450 2A4	Cyp2a4	-2.65	2.95	+	+	-2.64	-2.67	3.22	2.69
Q91X75	Q91X75_MOUSE	Cyp2a4 protein	Cyp2a5	-3.89	3.07	+	+	-4.03	-3.74	2.91	3.24
P33267	CP2F2_MOUSE	Cytochrome P450 2F2	Cyp2f2	-1.30	1.96	+	+	-1.40	-1.21	2.01	1.90
Q9WUZ9	ENTP5_MOUSE	Ectonucleoside triphosphate diphosphohydrolase 5	Entpd5	-2.06	3.94	+	+	-2.34	-1.79	3.72	4.17
Q9D379	HYEP_MOUSE	Epoxide hydrolase 1	Ephx1	-2.19	3.30	+	+	-2.41	-1.97	3.77	2.84
E9PV24	FIBA_MOUSE	Fibrinogen alpha chain	Fga	0.88	1.95	+	+	1.00	0.76	2.19	1.70
P15105	GLNA_MOUSE	Glutamine synthetase	Glul	1.08	2.04	+	+	1.04	1.13	2.48	1.60
P10649	GSTM1_MOUSE	Glutathione S-transferase Mu 1	Gstm1*	-3.37	2.93	+	+	-3.36	-3.38	2.66	3.21
Q8CFX1	G6PE_MOUSE	GDH/6PGL endoplasmic bifunctional protein	H6pd*	-0.82	2.39	+	+	-0.75	-0.88	2.43	2.35
Q8VCR2	DHB13_MOUSE	17-beta-hydroxysteroid dehydrogenase 13	Hsd17b13	-6.58	4.22	+	+	-6.95	-6.22	5.52	2.92
Q3U816	Q3U816_MOUSE	Oxidoreductase HTATIP2	Htatip2*	-3.39	4.21	+	+	-4.02	-2.76	4.71	3.71
Q9DCY0	KEG1_MOUSE	Glycine N-acyltransferase-like protein Keg1	Keg1	-1.34	1.97	+	+	-1.39	-1.29	2.15	1.79
Q71RI9	KAT3_MOUSE	Kynurenine--oxoglutarate transaminase 3	Kyat3	-0.84	2.95	+	+	-0.91	-0.77	3.06	2.83
Q7TNG8	LDHD_MOUSE	Probable D-lactate dehydrogenase, mitochondrial	Ldhd	-0.92	2.68	+	+	-1.02	-0.82	3.19	2.17
Q9DBN5	LONP2_MOUSE	Lon protease homolog 2, peroxisomal	Lonp2	-0.78	2.93	+	+	-0.86	-0.71	3.18	2.67
Q3ULD5	MCCB_MOUSE	Methylcrotonoyl-CoA carboxylase beta chain, mitochondrial	Mccc2	-0.56	2.74	+	+	-0.61	-0.50	2.71	2.76
Q8BH59	CMC1_MOUSE	Calcium-binding mitochondrial carrier protein Aralar1	Slc25a12	0.74	2.54	+	+	0.86	0.61	2.68	2.39
K9J7B2	K9J7B2_MOUSE	UDP-glucuronosyltransferase	Ugt1a6b	-1.20	2.20	+	+	-1.30	-1.10	2.30	2.09
Q8R084	Q8R084_MOUSE	UDP-glucuronosyltransferase	Ugt2b1	-1.39	3.66	+	+	-1.50	-1.28	3.15	4.17
Q8BJL9	Q8BJL9_MOUSE	UDP-glucuronosyltransferase	Ugt2b35*	-3.76	3.34	+	+	-3.69	-3.82	2.24	4.45
Q8K169	Q8K169_MOUSE	UDP-glucuronosyltransferase	Ugt2b5	-1.60	3.57	+	+	-1.63	-1.57	3.58	3.55

Table S1. Shortlist of 32 proteins identified as significantly different (FDR 10% & S0=0.1 - See Figure S1) in both MS runs from the liver samples. Student's two tailed t-test results and log_2 Nrf2 KO/Keap1 KD ratios are presented. A negative log_2 ratio is indicative of a protein whose abundance is positively influenced by Nrf2 and/or negatively influenced by Keap1. For further details see Data S1. Proteins organised by gene name. *Proteins also shortlisted from the organoid samples are marked with an asterisk. Related to Figure 1.

Intestinal Organoids

Uniprot Accession	Uniprot Locus	Protein name	Gene name	Average log_2 Nrf2 KO/Keap1 KD	Average -log_{10} p-value Nrf2 KO v Keap1 KD	T-test Significant Nrf2 KO v Keap KD Run 1	T-test Significant Nrf2 KO v Keap KD Run 2	log_2 Nrf2/Keap 1 Run 1	log_2 Nrf2/Keap 1 Run 2	-log p-value Nrf2 KO v Keap1 KD Run 1	-log p-value Nrf2 KO v Keap1 KD Run 2
B2RX12	MRP3_MOUSE	Canalicular multispecific organic anion transporter 2	Abcc3	-2.73	2.46	+	+	-3.08	-2.38	2.63	2.30
E9Q236	E9Q236_MOUSE	ATP-binding cassette, sub-family C (CFTR/MRP), member 4	Abcc4	-3.49	2.91	+	+	-3.01	-3.96	2.54	3.29
Q9QXD1	ACOX2_MOUSE	Peroxisomal acyl-coenzyme A oxidase 2	Acox2	-5.66	3.27	+	+	-5.61	-5.70	2.12	4.42
Q99NF1	BCDO2_MOUSE	Beta,beta-carotene 9,10-oxygenase	Bco2	2.34	2.63	+	+	3.15	1.53	2.95	2.32
P48758	CBR1_MOUSE	Carbonyl reductase [NADPH] 1	Cbr1*	-2.69	3.30	+	+	-2.73	-2.66	3.19	3.42
Q8K354	CBR3_MOUSE	Carbonyl reductase [NADPH] 3	Cbr3	-6.36	3.47	+	+	-6.06	-6.66	3.85	3.08
Q8VCC2	EST1_MOUSE	Liver carboxylesterase 1	Ces1*	-6.41	3.61	+	+	-5.91	-6.91	3.31	3.91
Q91WU0	CES1F_MOUSE	Carboxylesterase 1F	Ces1f*	-6.33	2.56	+	+	-6.70	-5.97	2.83	2.30
O88668	CREG1_MOUSE	Protein CREG1	Creg1*	-1.70	1.93	+	+	-1.64	-1.76	2.06	1.81
Q9WUD0	Q9WUD0_MOUSE	Cytochrome P450 2B10	Cyp2b10	2.40	1.90	+	+	2.39	2.41	2.00	1.81
O88533	DDC_MOUSE	Aromatic-L-amino-acid decarboxylase	Ddc	-3.24	2.29	+	+	-3.44	-3.05	2.31	2.27
Q9D379	HYEP_MOUSE	Epoxide hydrolase 1	Ephx1*	-1.63	2.66	+	+	-1.67	-1.60	2.78	2.54
Q8R180	ERO1A_MOUSE	ERO1-like protein alpha	Ero1a	-2.99	2.65	+	+	-3.21	-2.77	2.77	2.54
Q9D6U8	F162A_MOUSE	Protein FAM162A	Fam162a	-1.59	3.02	+	+	-1.69	-1.50	3.14	2.90
Q00612	G6PD1_MOUSE	Glucose-6-phosphate 1-dehydrogenase X	G6pdx	-2.72	2.99	+	+	-2.83	-2.61	3.31	2.67
P97494	GSH1_MOUSE	Glutamate--cysteine ligase catalytic subunit	Gclc	-4.97	3.29	+	+	-4.41	-5.53	2.84	3.75
O09172	GSH0_MOUSE	Glutamate--cysteine ligase regulatory subunit	Gclm	-2.92	3.28	+	+	-2.86	-2.97	2.90	3.66
Q64521	GPDM_MOUSE	Glycerol-3-phosphate dehydrogenase, mitochondrial	Gpd2	-0.84	2.89	+	+	-0.76	-0.92	2.98	2.81
A0A0R4J111	A0A0R4J111_MOUSE	Glutathione peroxidase	Gpx2	-2.86	2.97	+	+	-2.59	-3.13	3.36	2.58
P47791	GSHR_MOUSE	Glutathione reductase, mitochondrial	Gsr	-2.38	2.89	+	+	-2.44	-2.33	3.56	2.22
P13745	GSTA1_MOUSE	Glutathione S-transferase A1	Gsta1	-5.98	2.86	+	+	-7.23	-4.73	3.89	1.83
P30115	GSTA3_MOUSE	Glutathione S-transferase A3	Gsta3	-5.51	2.68	+	+	-5.91	-5.11	3.14	2.21
Q9DCM2	GSTK1_MOUSE	Glutathione S-transferase kappa 1	Gstk1	-1.04	2.26	+	+	-1.05	-1.03	2.37	2.14
P10649	GSTM1_MOUSE	Glutathione S-transferase Mu 1	Gstm1*	-5.02	3.76	+	+	-5.04	-5.00	3.74	3.78
P19639	GSTM3_MOUSE	Glutathione S-transferase Mu 3	Gstm3	-3.74	3.19	+	+	-3.68	-3.81	3.28	3.10
Q8CFX1	G6PE_MOUSE	GDH/6PGL endoplasmic bifunctional protein	H6pd*	-2.90	2.77	+	+	-2.96	-2.83	2.95	2.59
Q8K2C9	HACD3_MOUSE	Very-long-chain (3R)-3-hydroxyacyl-CoA dehydratase 3	Hacd3	-0.89	2.32	+	+	-0.82	-0.97	2.35	2.28
G3UVV4	G3UVV4_MOUSE	Hexokinase 1, isoform CRA_f	Hk1	-2.15	2.06	+	+	-2.14	-2.17	2.10	2.02
Q3U816	Q3U816_MOUSE	Oxidoreductase HTATIP2	Htatip2*	-2.16	2.80	+	+	-2.23	-2.08	3.78	1.83
F8VPT3	F8VPT3_MOUSE	Lactase	Lct	5.67	1.87	+	+	5.70	5.63	1.88	1.85
P06801	MAOX_MOUSE	NADP-dependent malic enzyme	Me1	-4.13	2.99	+	+	-4.22	-4.04	3.74	2.23
Q91VS7	MGST1_MOUSE	Microsomal glutathione S-transferase 1	Mgst1	-0.99	2.08	+	+	-1.02	-0.97	2.09	2.06
Q7M758	NALDL_MOUSE	Aminopeptidase NAALADL1	Naaladl1	-3.94	2.43	+	+	-4.10	-3.79	3.01	1.85
Q64669	NQO1_MOUSE	NAD(P)H dehydrogenase [quinone] 1	Nqo1	-5.28	3.90	+	+	-5.25	-5.31	4.30	3.49
Q9DCD0	6PGD_MOUSE	6-phosphogluconate dehydrogenase, decarboxylating	Pgd	-1.49	3.19	+	+	-1.62	-1.35	3.05	3.32
Q9D711	PIR_MOUSE	Pirin	Pir	-2.29	1.94	+	+	-2.37	-2.20	2.09	1.79
Q9DBX5	Q9DBX5_MOUSE	Phospholipase A2	Pla2g4a	-1.82	1.87	+	+	-1.59	-2.05	1.83	1.90
Q91YR9	PTGR1_MOUSE	Prostaglandin reductase 1	Ptgr1	-2.97	2.71	+	+	-3.14	-2.79	3.34	2.07
P43137	LIT1_MOUSE	Lithostathine-1	Reg1	3.55	2.50	+	+	3.56	3.54	2.70	2.30
P52760	RIDA_MOUSE	2-iminobutanoate/2-iminopropanoate deaminase	Rida	-4.30	2.98	+	+	-4.16	-4.45	2.80	3.15
Q99P72	RTN4_MOUSE	Reticulon-4	Rtn4	-1.38	2.35	+	+	-1.36	-1.40	2.03	2.67
Q91Y74	SIA4C_MOUSE	CMP-N-acetylneuraminate-beta-galactosamide-alpha-2,3-sialyltransferase 4	St3gal4	6.31	3.90	+	+	6.20	6.43	3.58	4.21
Q9D939	ST1C2_MOUSE	Sulfotransferase 1C2	Sult1c2	2.05	2.48	+	+	1.87	2.23	2.17	2.80
E9Q6Q8	E9Q6Q8_MOUSE	TBC1 domain family member 4	Tbc1d4	-1.55	2.07	+	+	-1.30	-1.80	2.18	1.95
Q9JMH6	TRXR1_MOUSE	Thioredoxin reductase 1, cytoplasmic	Txnrd1	-1.46	2.50	+	+	-1.28	-1.63	2.99	2.00
O70475	UGDH_MOUSE	UDP-glucose 6-dehydrogenase	Ugdh	-3.28	3.40	+	+	-3.32	-3.23	3.59	3.20
Q91ZJ5	UGPA_MOUSE	UTP--glucose-1-phosphate uridylyltransferase	Ugp2	-2.04	2.31	+	+	-1.82	-2.25	1.82	2.79
Q64435	UD16_MOUSE	UDP-glucuronosyltransferase 1-6	Ugt1a6	-3.37	2.38	+	+	-3.37	-3.36	2.11	2.66
Q8BJL9	Q8BJL9_MOUSE	UDP-glucuronosyltransferase	Ugt2b35*	-2.32	3.57	+	+	-2.44	-2.20	3.99	3.15
Q3UEP4	Q3UEP4_MOUSE	UDP-glucuronosyltransferase	Ugt2b36	-1.99	2.20	+	+	-1.98	-2.00	1.79	2.61

Table S2. Shortlist of 50 proteins identified as significantly different (FDR 10% & S0=0.1 - See Figure S1) in both MS runs from the intestinal organoid samples. Student's two tailed t-test results and log_2 Nrf2 KO/Keap1 KD ratios are presented. A negative log_2 ratio is indicative of a protein whose abundance is positively influenced by Nrf2 and/or negatively influenced by Keap1. For further details see Data S1. Proteins organised by gene name. *Proteins also shortlisted from the liver samples are marked with an asterisk. Related to Figure 1.

Gene name	Fasta headers	Gluconeogenesis related	Liver_log2 NRF2/KEAP 1	Liver_p-value NRF2/KEAP 1	Student's T-test Significant Liver	Organoids_log2 NRF2/KEAP 1	Organoids_p-value NRF2/KEAP 1	Student's T-test Significant Organoids
Aldoa	sp|P05064|ALDOA_MOUSE Fructose-bisphosphate aldo	+	0.21	0.28		-0.31	1.01	
Aldob	sp|Q91Y97|ALDOB_MOUSE Fructose-bisphosphate ald	+	0.46	0.65		0.68	0.67	
Aldoc	sp|P05063|ALDOC_MOUSE Fructose-bisphosphate aldo	+				0.64	1.23	
Dera	sp|Q91YP3|DEOC_MOUSE Deoxyribose-phosphate aldo	+				0.51	1.42	
Eno1	sp|P17182|ENOA_MOUSE Alpha-enolase OS=Mus mus	+	0.56	0.88		0.46	0.47	
Fbp1	sp|Q9QXD6|F16P1_MOUSE Fructose-1,6-bisphosphata	+	1.52	1.10		1.77	1.61	
Fbp2	sp|P70695|F16P2_MOUSE Fructose-1,6-bisphosphatas	+				1.05	1.21	
G6pc	sp|P35576|G6PC_MOUSE Glucose-6-phosphatase OS=I	+	1.09	0.65				
Gapdh	sp|P16858|G3P_MOUSE Glyceraldehyde-3-phosphate d	+	0.65	0.61		0.30	1.17	
Got1	sp|P05201|AATC_MOUSE Aspartate aminotransferase,	+	1.14	0.76		1.18	1.11	
Got2	sp|P05202|AATM_MOUSE Aspartate aminotransferase	+	0.29	0.91		-0.33	0.36	
Gpd1	sp|P13707|GPDA_MOUSE Glycerol-3-phosphate dehydr	+	-0.27	1.86		0.77	0.73	
Gpd2	sp|Q64521|GPDM_MOUSE Glycerol-3-phosphate dehyd	+	0.21	0.54		-0.84	2.89	+
Gpi	sp|P06745|G6PI_MOUSE Glucose-6-phosphate isomera	+				0.32	0.25	
Hk1	tr|G3UVV4|G3UVV4_MOUSE Hexokinase 1, isoform CR	+	-1.50	0.66		-2.15	2.06	+
Hk2	sp|O08528|HXK2_MOUSE Hexokinase-2 OS=Mus muscu	+				-1.15	1.60	
Hkdc1	sp|Q91W97|HKDC1_MOUSE Hexokinase HKDC1 OS=Mu	+				-0.66	0.95	
Hoga1	sp|Q9DCU9|HOGA1_MOUSE 4-hydroxy-2-oxoglutarate	+	-0.06	0.12				
Khk	tr|A0A0J9YU79|A0A0J9YU79_MOUSE Ketohexokinase (	+				1.77	1.03	
Mdh1	sp|P14152|MDHC_MOUSE Malate dehydrogenase, cyto	+	-0.09	0.30		0.31	0.51	
Mdh2	sp|P08249|MDHM_MOUSE Malate dehydrogenase, mit	+	-0.11	0.37		-0.09	0.32	
Pck2	sp|Q8BH04|PCKGM_MOUSE Phosphoenolpyruvate carb	+	-1.81	0.29		-0.91	0.52	
Pcx	tr|G5E8R3|G5E8R3_MOUSE Pyruvate carboxylase OS=I	+	-0.49	1.44		-0.95	1.13	
Pgam1	sp|Q9DBJ1|PGAM1_MOUSE Phosphoglycerate mutase	+	1.06	1.16		0.40	0.63	
Pgk1	sp|P09411|PGK1_MOUSE Phosphoglycerate kinase 1 O	+	0.93	0.61		0.42	0.94	
Pgm1	sp|Q9D0F9|PGM1_MOUSE Phosphoglucomutase-1 OS=	+	1.16	0.89		-1.05	1.84	
Rbp4	sp|Q00724|RET4_MOUSE Retinol-binding protein 4 OS	+	0.38	0.78				
Slc25a1	sp|Q8JZU2|TXTP_MOUSE Tricarboxylate transport prot	+	-0.18	0.98		-0.51	1.10	
Slc25a10	sp|Q9QZD8|DIC_MOUSE Mitochondrial dicarboxylate ca	+	-0.22	0.82		-0.21	0.61	
Slc25a11	sp|Q9CR62|M2OM_MOUSE Mitochondrial 2-oxoglutara	+	0.15	0.62		0.04	0.03	
Slc25a12	sp|Q8BH59|CMC1_MOUSE Calcium-binding mitochondr	+	0.74	2.54		0.08	0.12	
Slc25a13	sp|Q9QXX4|CMC2_MOUSE Calcium-binding mitochondr	+	-0.15	0.50		-0.16	0.16	
Slc37a4	tr|Q9D1F9|Q9D1F9_MOUSE Solute carrier family 37 (G	+	-0.32	0.24		1.57	1.25	
Taldo1	sp|Q93092|TALDO_MOUSE Transaldolase OS=Mus mus	+				-0.41	1.74	
Tpi1	sp|P17751|TPIS_MOUSE Triosephosphate isomerase O	+	0.80	0.40		0.76	0.70	

Table S3. Proteins related to gluconeogenesis that were detected in both MS runs from the liver and organoid samples (see also Figure S4). Student's two tailed t-test was used to determine statistical significance, and log2 Nrf2 KO/Keap1 KD ratios are presented. A negative log2 ratio is indicative of a protein whose abundance is positively influenced by Nrf2 and/or negatively influenced by Keap1. Related to Figure 3.

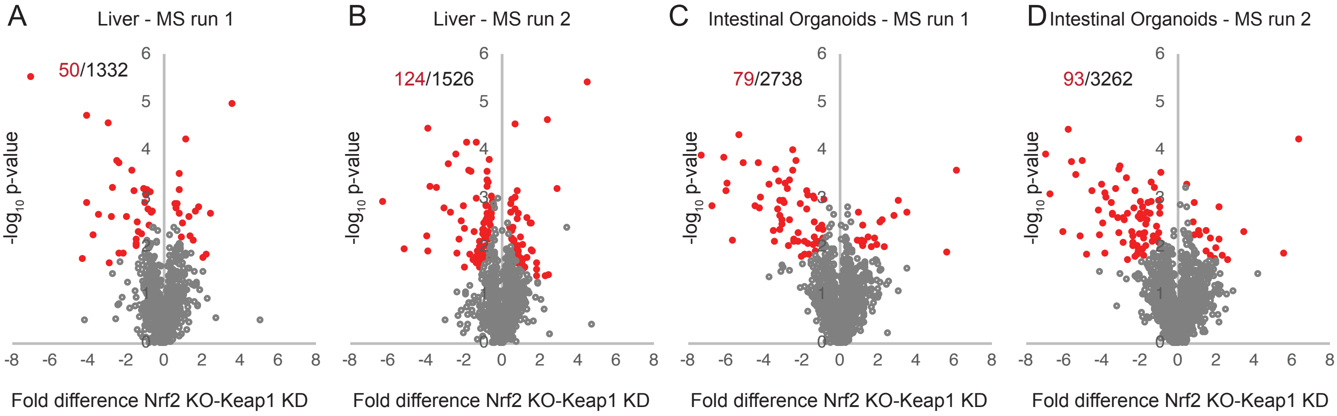

Figure S1. Proteomic analyses of mitochondria-enriched preparations from liver and early-passage intestinal organoids from wild-type (WT), Nrf2-knockout (Nrf2-KO), and Keap1-knockdown (Keap1-KD) mice. Fold change versus t-test p-value results for proteins quantified in each MS run for liver (A,B) and intestinal organoids (C,D). Red markers are those proteins that met the statistical cutoff of 10% FDR and S0=0.1 in two samples unpaired Student's t-test. See Data S1 for details. Related to Figure 1.

Liver

STRING protein interaction networks

A **Protein processing in endoplasmic reticulum and signal peptidase complex**

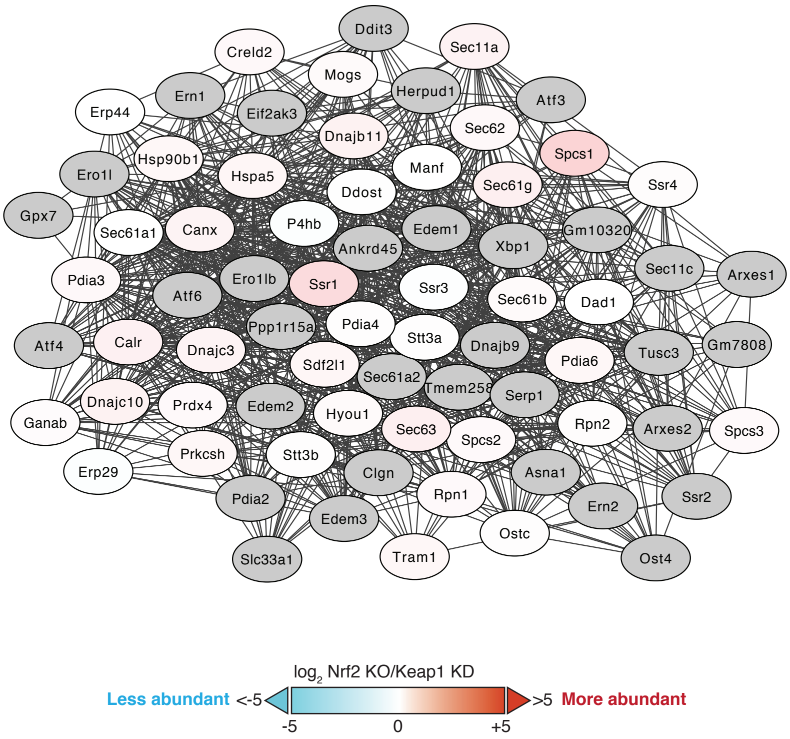

B

Mitochondrial Complex I biogenesis

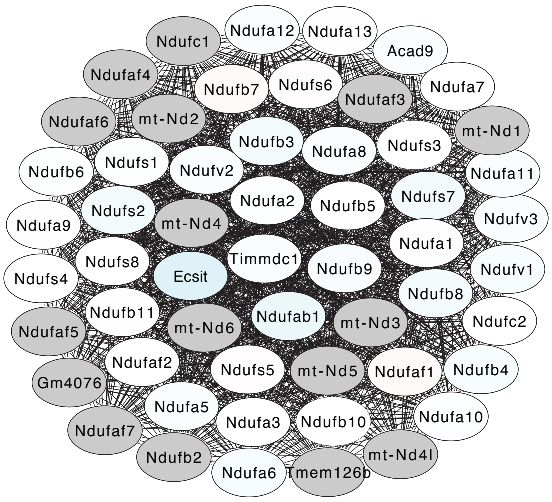

Figure S2. Network of proteins identified by STRING with both functional enrichments and quantitative relationships in mitochondria-enriched preparations from livers of Nrf2-knockout (Nrf2-KO) and Keap1-knock-down (Keap1-KD) mice. In addition to clusters of metabolic proteins within the Ces1 and Ugt families shown in Figure 2A, this type of analysis identified proteins with roles in protein processing in endoplasmic reticulum and signal peptidase complex (A), and proteins involved in mitochondrial complex I biogenesis (B) as statistically significantly different between the Nrf2-KO and Keap1-KD genotypes. Related to Figure 2.

Intestinal organoids STRING protein interaction networks

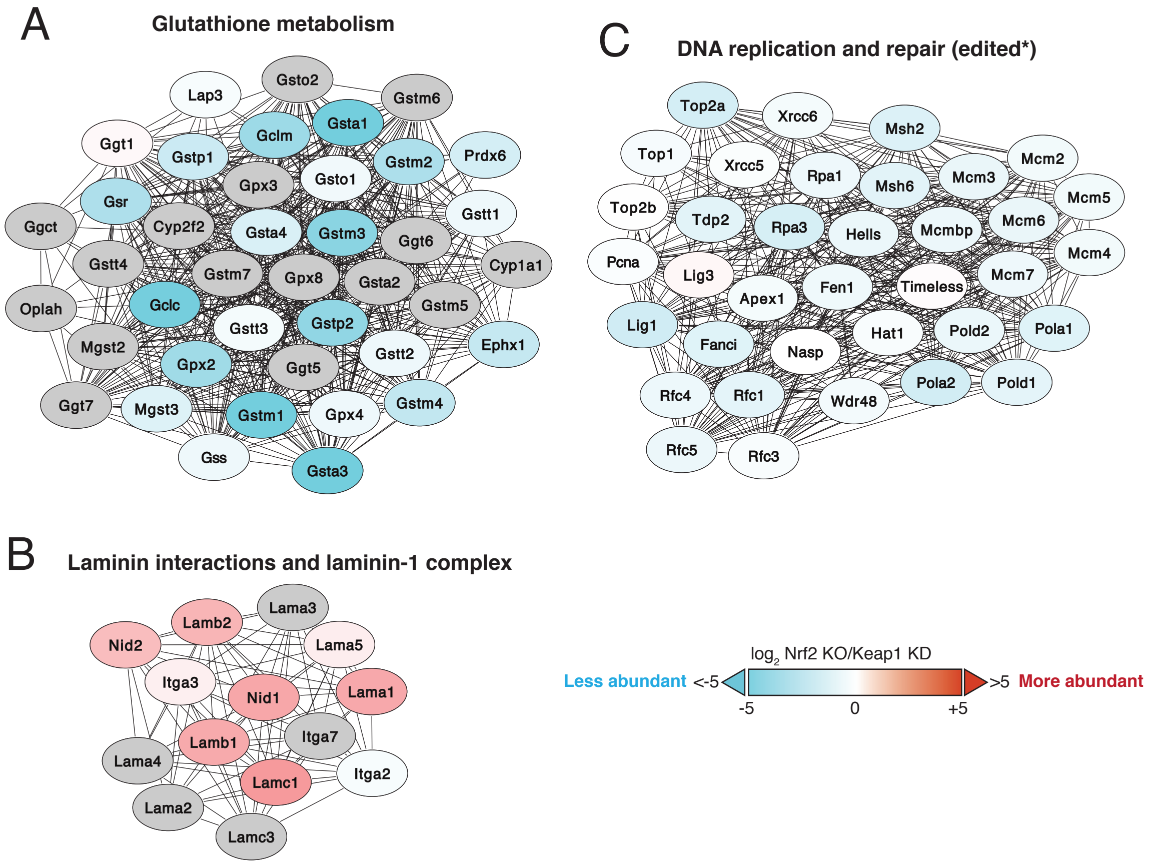

Figure S3. Network of proteins identified by STRING with both functional enrichments and quantitative relationships in mitochondria-enriched preparations from early-passage intestinal organoids from Nrf2-knockout (Nrf2-KO) and Keap1-knockdown (Keap1-KD) mice. In addition to clusters of metabolic proteins within the Ces1 and Cyp families shown in Figure 2B, and proteins involved in glycolysis and the pentose phosphate pathway shown in Figure 3A, this type of analysis identified glutathione metabolism (A), extracellular matrix proteins (B), and a group of DNA replication and repair proteins (C) as statistically significantly different between the Nrf2-KO and Keap1-KD genotypes. Related to Figures 2 and 3.

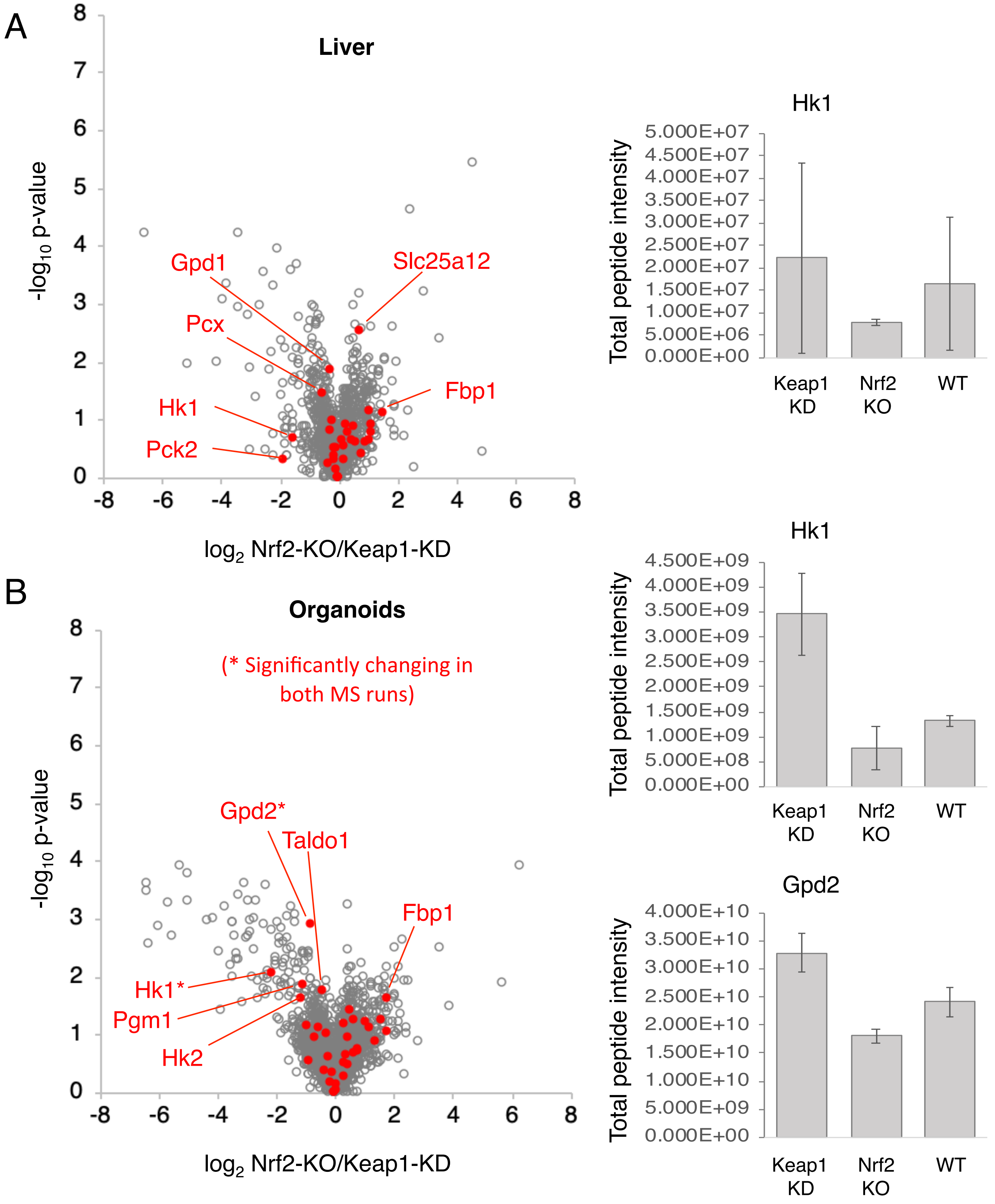

Figure S4. Proteomic analyses of enzymes related to gluconeogenesis in mitochondria-enriched preparations from liver and early-passage intestinal organoids from wild-type (WT), Nrf2-knockout (Nrf2-KO), and Keap1-knockdown (Keap1-KD) mice. Fold change versus t-test p-value results for proteins quantified in the two MS runs for liver (A) and intestinal organoids (B). Red markers are proteins related to gluconeogenesis. Those that met the statistical cutoff of 10% FDR and S0=0.1 in two samples unpaired Student's t-test are indicated with an asterisks. The side panels show the protein intensities for Hk1 (liver), and for Hk1 and Gpd2 (organoids) in the three genotypes. Related to Figure 3.

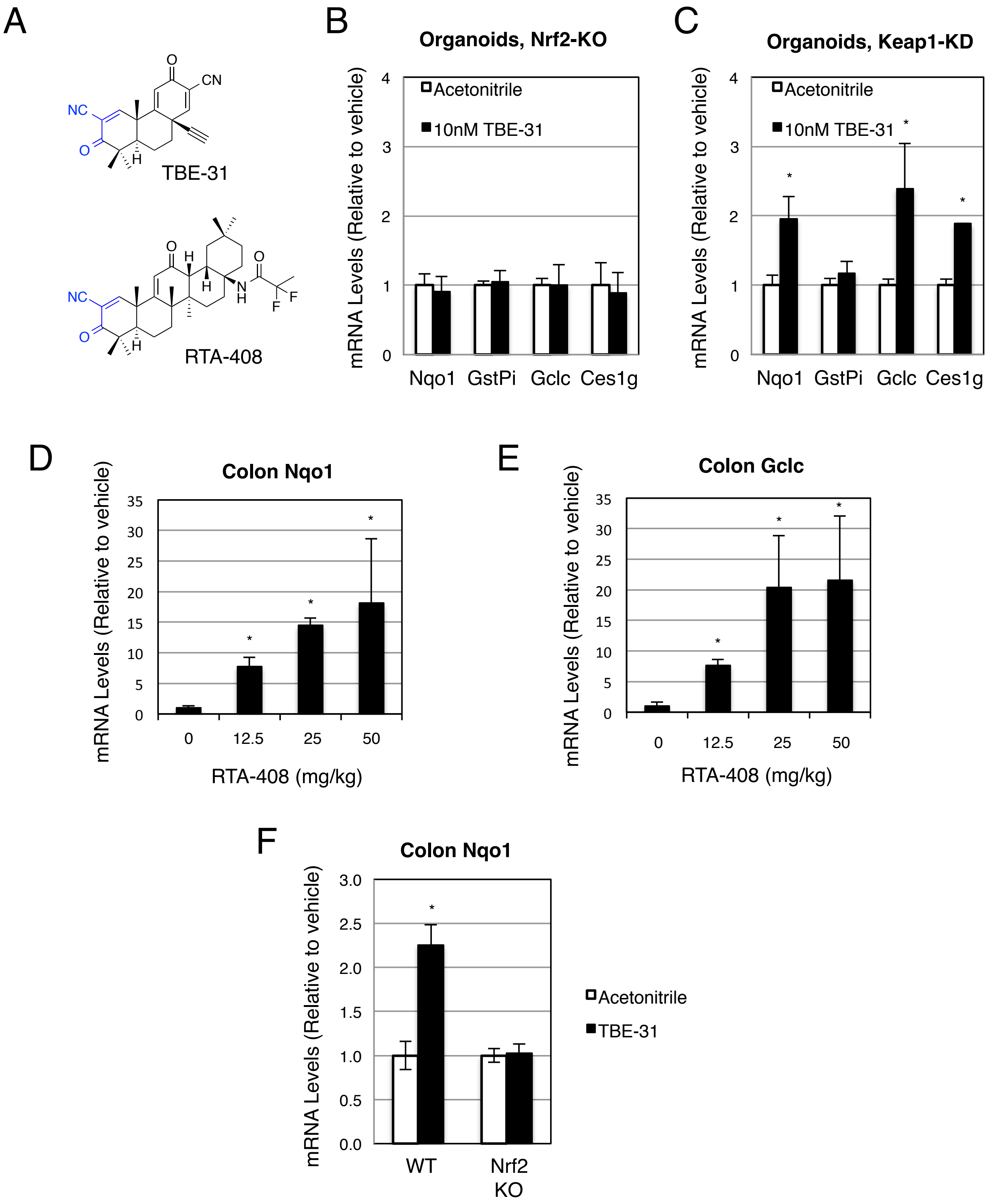

Figure S5. The cyclic cyanoenones TBE-31 and RTA-408 activate Nrf2-dependent transcription. (A) Chemical structures of TBE-31 and RTA-408. (B,C) mRNA levels for Nqo1, Gstp, Gclc and Ces1g in intestinal organoids from Nrf2-knockout (Nrf2-KO) (B) and Keap1-knockdown (Keap1-KD) (C) mice. The organoids (n=3) were treated with the Nrf2 activator TBE-31 (10 nM, 16h) or vehicle (0.1% acetonitrile). (D,E) mRNA levels for Nqo1 (D) and Gclc (E) in colons of male WT C57BL6 mice (n=3-4) that had been treated with RTA-408, *per os*, 3 times, 24h-apart, and colon tissue was harvested 6h after the last dose. (F) mRNA levels for Nqo1 in colons of male C57BL6 wild type (WT) and Nrf2-knockout (Nrf2-KO) mice (n=4-5). The animals were treated with TBE-31 (5 nmol/g body weight, 3 times, at 24-h intervals, *per os,* black bars) or vehicle (1% DMSO in corn oil, white bars), and fasted for 4h before tissue harvesting. Related to Figure 4.

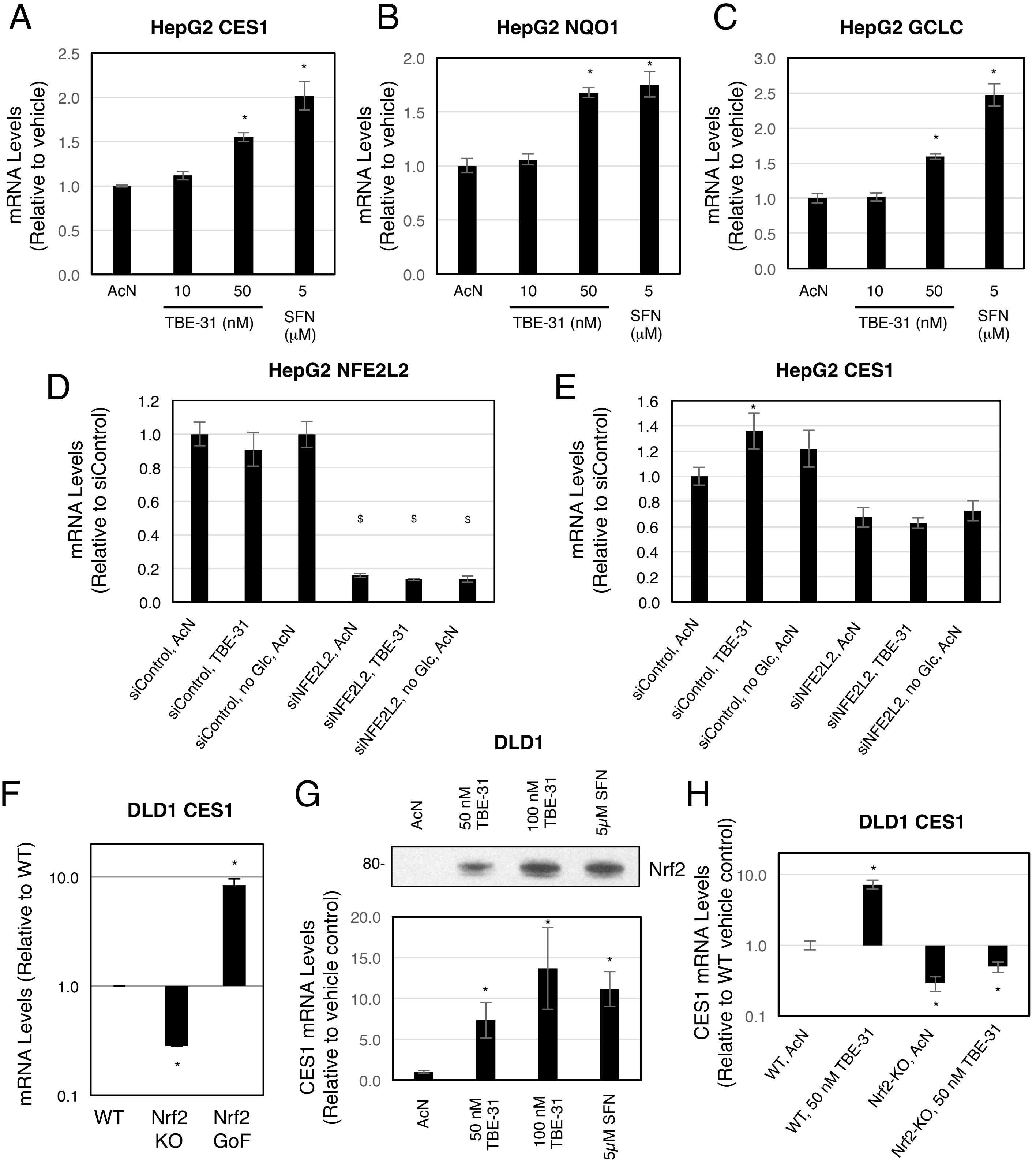

Figure S6. Nrf2 dependence of human CES1 expression. (A-C) mRNA levels for CES1 (A), NQO1 (B) and GCLC (C) in HepG2 cells following 16h treatment with Nrf2 activators TBE-31 (10 and 50 nM), SFN (5 μM) or vehicle (0.1% acetonitrile). (D-E) mRNA levels for *NFE2L2* (D) and CES1 (E) in HepG2 cells following siRNA knock-down of *NFE2L2* for 45h combined with TBE-31 (100nM, last 16h) or glucose deprivation (last 27h), or vehicle control treatment (0.1% acetonitrile). (F) mRNA levels for CES1 in isogenic DLD1 cell lines with either unaltered Nrf2 (WT), or Nrf2-knockout (Nrf2-KO) or Nrf2-gain-of-function (Nrf2-GoF) mutations. (G) Nrf2 protein (top panel) and CES1 mRNA levels (bottom panel) in DLD1 cells following 17h treatment with Nrf2 activators TBE-31 (50 and 100 nM), SFN (5 μM) or vehicle (0.1% acetonitrile). (H) CES1 mRNA levels in unaltered (WT) and Nrf2-knockout (Nrf2-KO) isogenic DLD1 cell lines following 17h treatment with Nrf2 activator TBE-31 (50 nM) or vehicle (0.1% acetonitrile). *p<0.01, relative to the respective leftmost control (A,B,C,E,G,H,F); $^{\$}p<0.01$, changes in response to si*NFE2L2* treatment (D). Related to Figure 4.

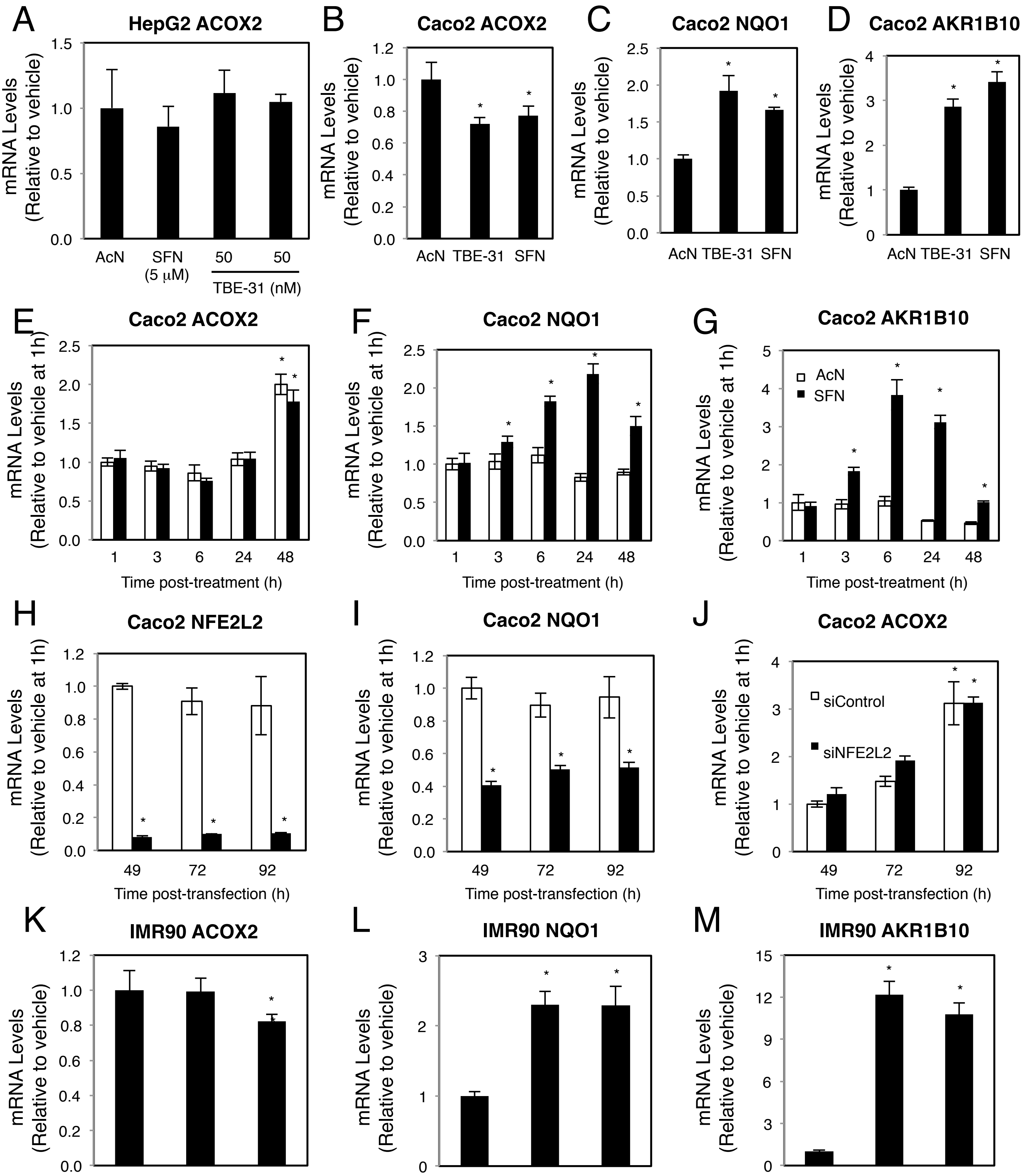

Figure S7. (A) mRNA levels for ACOX2 in HepG2 cells following 16h treatment with Nrf2 activators SFN, TBE-31, or vehicle (0.1% acetonitrile). (B-D) mRNA levels for ACOX2 (B), NQO1 (C) and AKR1B10 (D) in Caco2 cells following 16h treatment with Nrf2 activators TBE-31 (100 nM), SFN (5 μM) or vehicle in serum-free media. (E-G) mRNA levels for ACOX2 (E), NQO1 (F) and AKR1B10 (F) in Caco2 treated with the Nrf2 activator SFN (5 μM, black bars) or vehicle (white bars) for the indicated times. *p<0.05, compared to vehicle at the 1h-time point. (H-J) mRNA levels for *NFE2L2* (H), NQO1 (I) and ACOX2 (J) in Caco2 following transfection with siRNA targeting *NFE2L2* (black bars) or no-targeting si-control (white bars) for the indicated times. *p<0.05, compared to vehicle at the 49h-time point. (K-M) mRNA levels for ACOX2 (K), NQO1 (L) and AKR1B10 (M) in IMR90 cells following 16h treatment with Nrf2 activators SFN (5 μM), TBE-31 (100 nM), or vehicle. *p<0.05, compared to vehicle. Related to Figure 4.

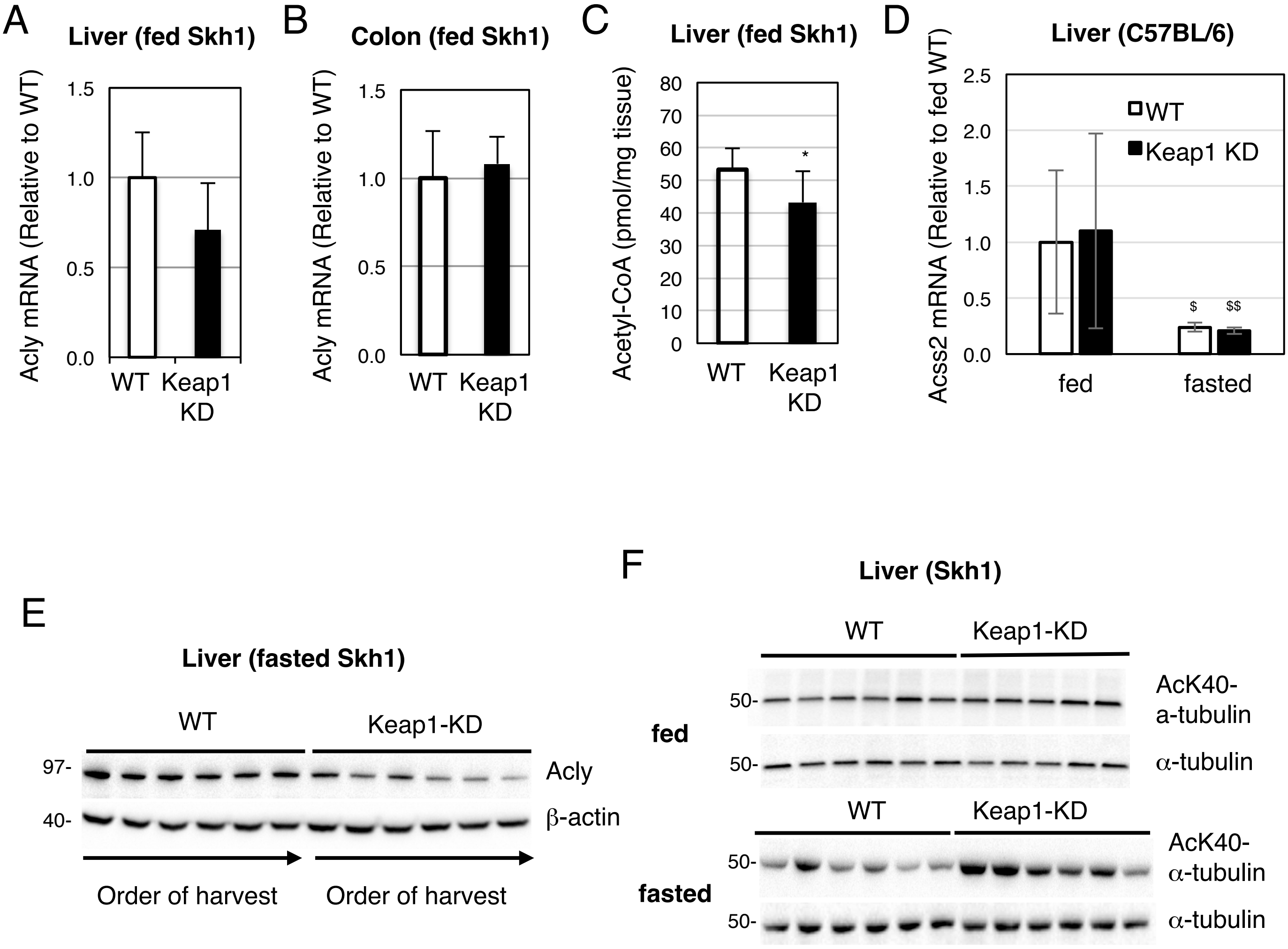

Figure S8. Fasting decreases the expression of hepatic Acly, Acss2 and the levels of acetyl-CoA. (A,B) mRNA levels for Acly in livers (A) and colons (B) from *ad libitum*-fed wild-type (WT) and Keap1-knockdown (Keap1-KD) female Skh1-hairelss mice (n=6); *18S* used as a reference gene. (C) Acetyl-CoA levels in livers from *ad libitum*-fed wild-type (WT) and Keap1-knockdown (Keap1-KD) female Skh1-hairelss mice (n=5-10); *p < 0.05. (D) mRNA levels for Acss2 in livers from wild-type (WT) and Keap1-knockdown (Keap1-KD) female C57BL/6 mice (n=8) that were either fed *ad libitum* or fasted for 18h; *18S* used as a reference gene; $^{\$}$p<0.01 and $^{\$\$}$p<0.05, compared to respective fed genotype. (E) Protein levels for Acly in livers of fasted wild-type (WT) and Keap 1-knockdown (Keap1-KD) female Skh1-hairless mice (n=6). (F) Levels of AcK40-α-tubulin and α-tubulin in livers of fed (top blot) or overnight-fasted (bottom blot) wild-type (WT) and Keap1-knockdown (Keap1-KD) Skh1-hairless mice (n=5-6). Related to Figures 6 and 7.

A

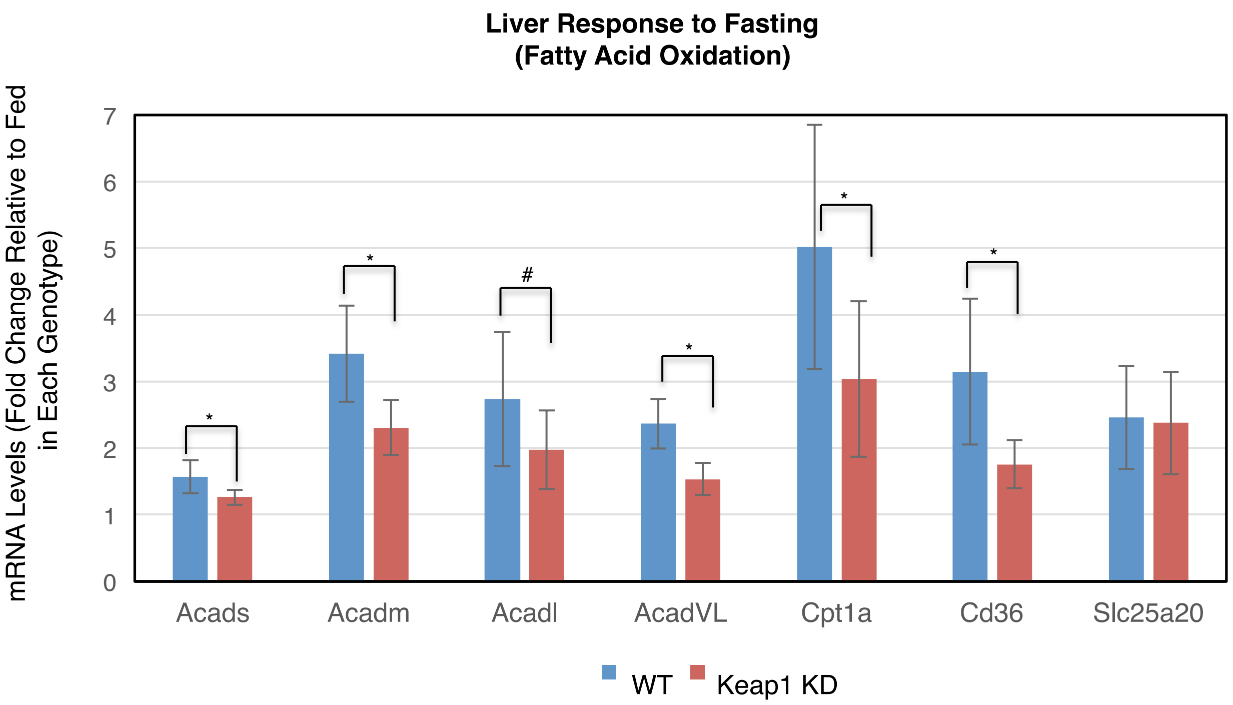

B

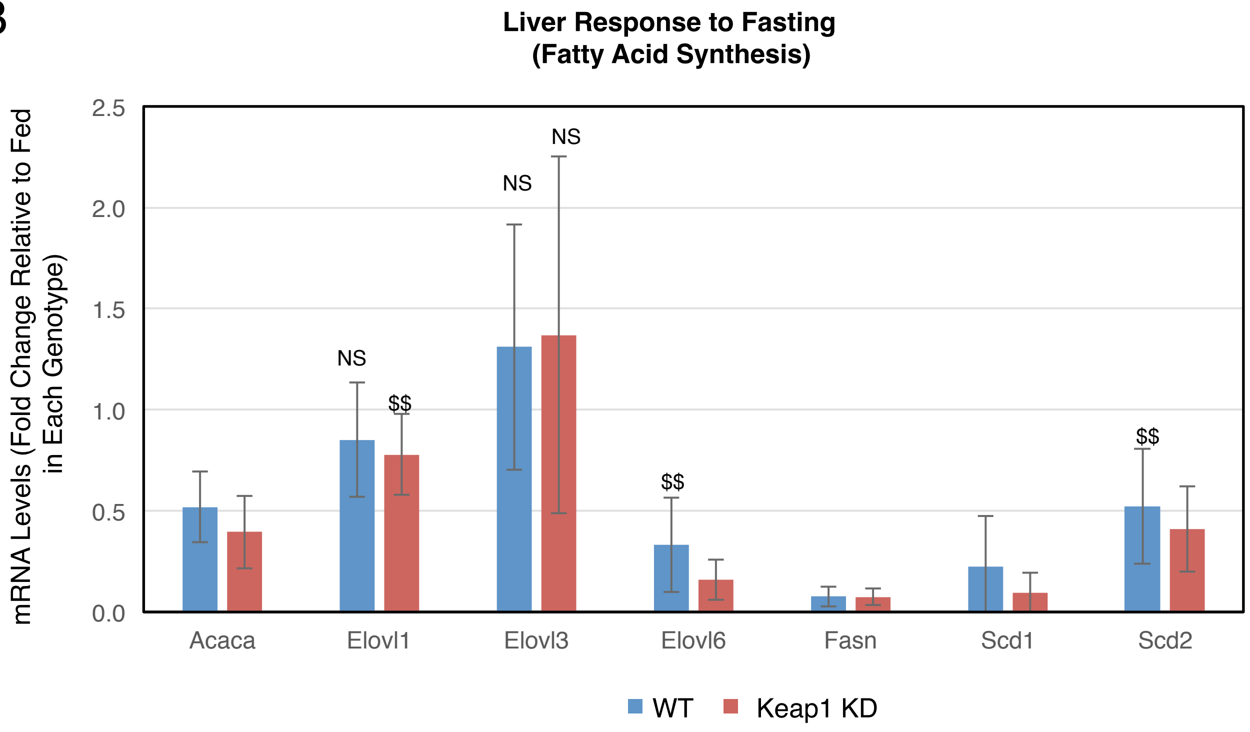

Figure S9. Liver response to fasting. Fasted-to-fed ratios of mRNA levels for proteins involved in FAO (A) and FAS (B) in livers from wild-type (WT) and Keap1-knockdown (Keap1-KD) female C57BL/6 mice (n=8) that were either fed *ad libitum* or fasted for 18h. *18S* used as a reference gene. Effect of fasting on gene expression was significant (p<0.01, not shown) for all FAO genes (A) and most FAS genes (B) except where marked: $^{\$\$}$0.01<p<0.05, ^{NS} – non-significant; significance of the genotype and feeding status interaction (Type I Anova): *p<0.01, $^{\#}$0.05<p<0.1. Related to Figure 6.

A

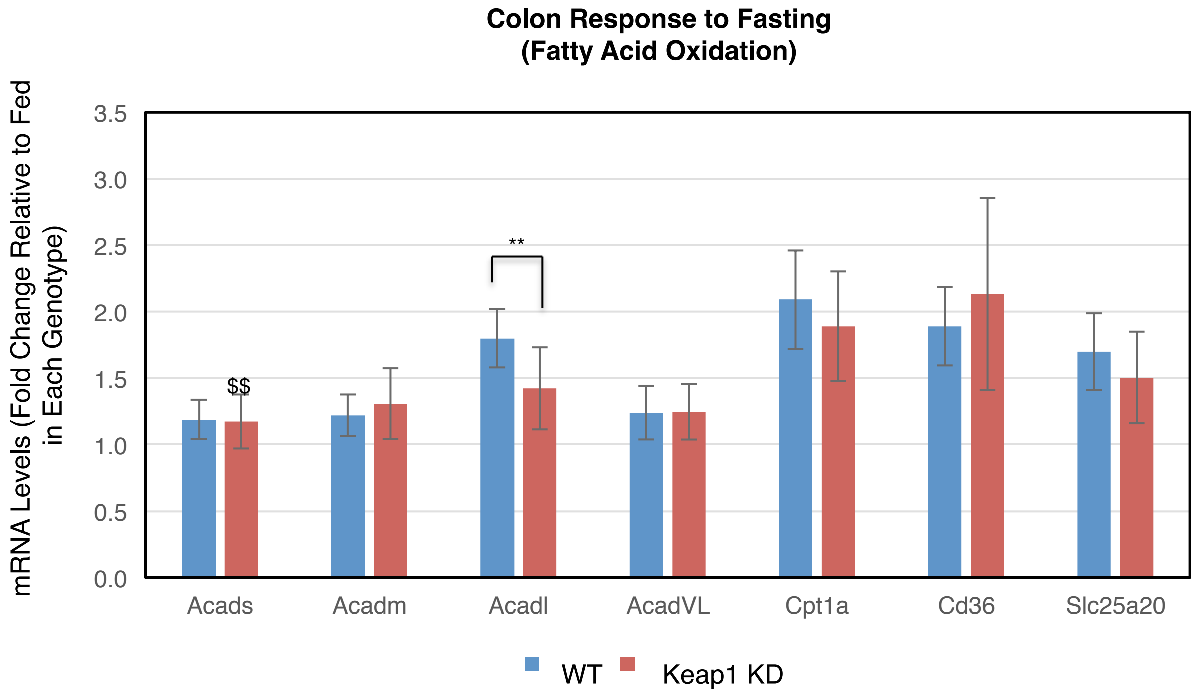

B

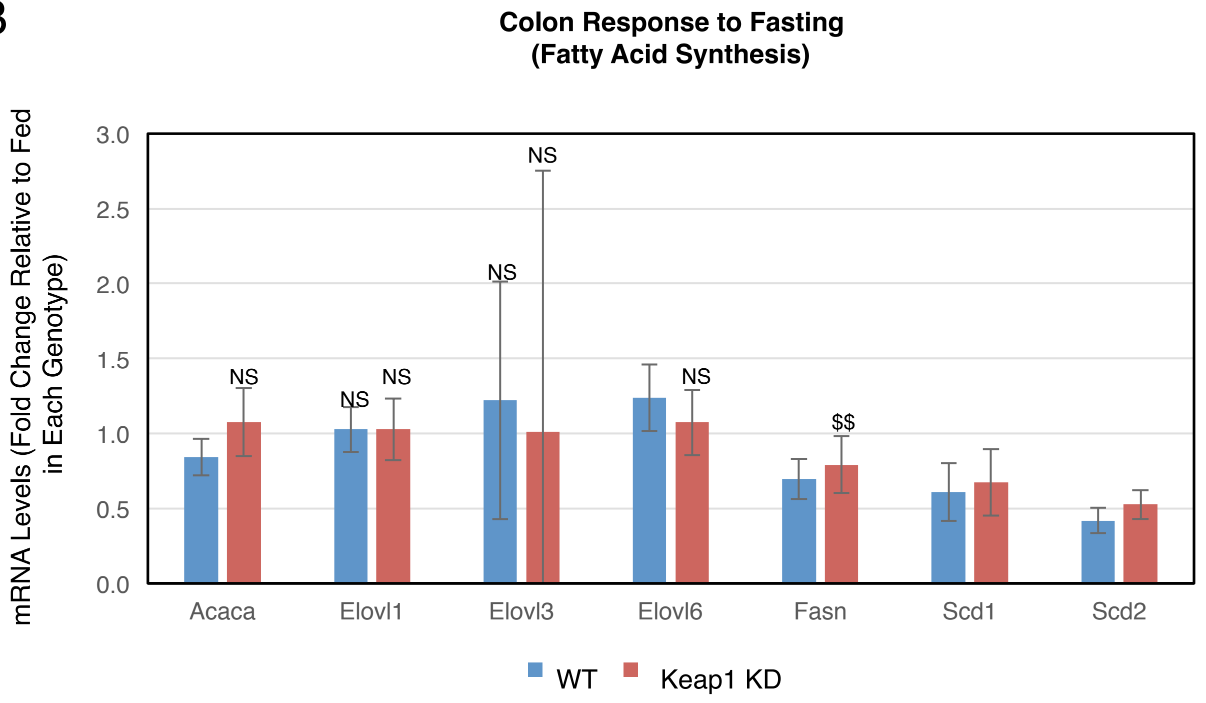

Figure S10. Colon response to fasting. Fasted-to-fed ratios of mRNA levels for proteins involved in FAO (A) and FAS (B) in colons from wild-type (WT) and Keap1-knockdown (Keap1-KD) female C57BL/6 mice (n=8) that were either fed *ad libitum* or fasted for 18h. *18S* used as a reference gene. Effect of fasting on gene expression was significant (p<0.01, not shown) for most FAO genes (A) and FAS genes (B) except where marked: $^{\$\$}$ 0.01<p<0.05, ^{NS} – non-significant; significance of the genotype and feeding status interaction (Type I Anova): **0.01<p<0.05. Related to Figure 6.

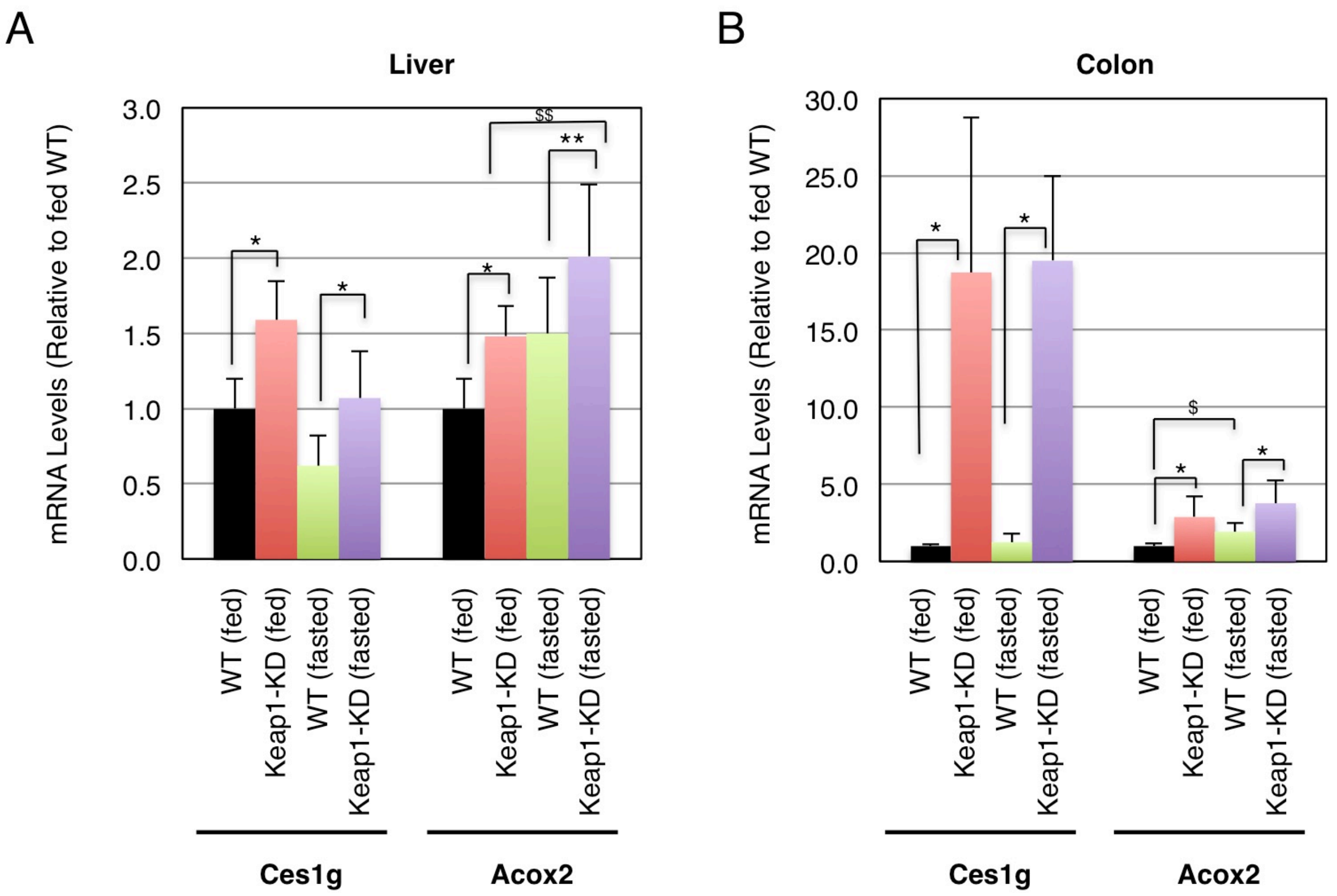

Figure S11. Downregulation of Keap1 increases the expression of Ces1g and Acox2 at fed and fasted states. mRNA levels for Ces1g and Acox2 in livers (A) and colons (B) from wild-type (WT) and Keap1-knockdown (Keap1-KD) female C57BL/6 mice (n=8) that were either fed *ad libitum* or fasted for 18h. *18S* used as a reference gene. Significance of the difference between the genotypes at the same feeding status: *p < 0.01, **0.01<p<0.05. Effect of fasting within each genotype in liver (A) was significant in all cases (p<0.01, not labelled, $$0.01<p<0.05). Effect of fasting within each genotype in colon (B) did not reach significance except for Acox2 in WT ($p < 0.01). Related to Figures 4 and 6.

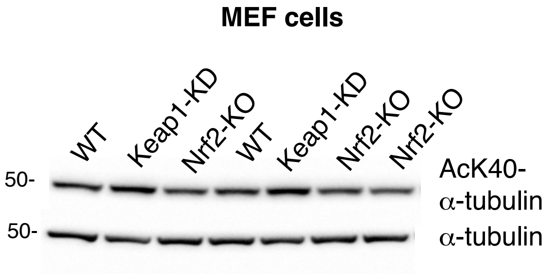

Figure S12. Downregulation of Keap1 increases, whereas depletion of Nrf2 decreases the acetylation of α-tubulin in mouse embryonic fibroblast (MEF) cells. Levels of AcK40-α-tubulin and α-tubulin in primary MEF cells from wild-type (WT), Nrf2-knockout (Nrf2-KO), and Keap1-knockdown (Keap1-KD) mice. Related to Figures 6 and 7.

TRANSPARENT METHODS

Materials

All chemicals and reagents were of the highest purity available and were purchased from common commercial suppliers. RTA-408 was from Cayman Chemical. *R,S*-sulforaphane (SFN) was from LKT Labs. (±)-TBE-31 was synthesized as described previously (Honda et al., 2011). For organoid culture experiments, a stock solution of TBE-31 in acetonitrile (AcN) was prepared and diluted (1:1,000) in the culture medium to achieve the final concentrations indicated in the figure legends. The final concentration of the solvent in the culture medium was 0.1% (v/v). For administration to animals by oral gavage, stock solutions of TBE-31 and RTA-408 in DMSO were prepared and diluted (1:100) in corn oil to achieve the compound doses indicated in the figure legends. The control mice received equal volumes of 1% DMSO in corn oil.

Antibodies

Solutions of primary antibodies were prepared in 5% (v/v) non-fat milk or 3% (w/v) BSA (anti-AcK40-α-tubulin only) in PBST. The following antibodies were used: rabbit polyclonal anti-carboxylesterase 1 (Ces1), 1:1,000, Abcam; rabbit monoclonal anti-acetylated (AcK40)-α-tubulin, 1:1,000, CST; mouse monoclonal anti-α-tubulin, 1:1,000, CST; rabbit polyclonal anti-ATP-citrate lyase (Acly), 1:1,000, CST; rabbit polyclonal anti-GAPDH, 1:5,000, Sigma; mouse monoclonal anti-β-actin, 1:10,000, Sigma; rabbit monoclonal LC3B, 1:1000, CST.

Animals

All mouse experiments were performed after ethical approval, in accordance with the regulations described in the UK Animals (Scientific Procedures) Act 1986. Mice were bred and maintained at the Medical School Resource Unit of the University of Dundee, with free access to water and food (pelleted RM1 diet from SDS Ltd., Witham, Essex, UK), on a 12-h light/ 12-h dark cycle, 35% humidity. Wild-type, Nrf2-knockout ($Nrf2^{-/-}$, N0) and Keap1-knockdown ($Keap1^{flox/flox}$, KD) mice (Taguchi et al., 2010) were originally generously provided by Masayuki Yamamoto (Tohoku University, Japan), and the resulting mouse colonies were maintained on either

C57BL/6 or Skh-1 hairless genetic backgrounds. Both male and female mice were used.

Isolation of mitochondria-enriched fractions from liver

To obtain mitochondria-enriched preparations from liver for proteomic analysis, 3 individual 20-22-week-old female mice of each genotype, all on the Skh-1 hairless genetic background, were used. The animals were euthanized by cervical dislocation, followed by decapitation and the blood was drained for 20 sec. All subsequent steps were performed on ice using pre-chilled solutions and instruments. Large bore pipette tips were used (made by cutting 2-3 mm off the standard tip end). Excised fresh whole livers were rinsed in 100 ml of ice-cold PBS and then in 50 ml of ice-cold Mitochondrial Isolation Buffer (MIB, 250 mM sucrose, 1mM EGTA, 20 mM Tris-Cl pH 7.4), transferred into a fresh beaker with 15 ml MIB on ice, chopped with scissors and then homogenised using Dounce homogeniser for 5 min on ice. Homogenates were clarified by centrifugation at 400 x *g* for 6 min (Sorvall T21, SL50T), and the resultant supernatants were transferred into fresh tubes and subjected to centrifugation, first at 1,200 *x* g for 6 min, followed by 8,000 x *g* for 10 min. The resulting pellets were gently re-suspended in 25 ml MIB, transferred into fresh tubes and subjected to centrifugation at 8,000 x *g* for 12 min. The supernatants were discarded and pellets washed twice by gently swirling the tube walls with 25 ml of MIB. The pellets were then carefully re-suspended in 1 ml MIB, frozen in aliquots in liquid nitrogen, and stored at -80°C until further use. Small aliquots of fractions from all purification steps were taken and analysed by immunoblotting for Vdac1, Lamin A and Actin B as quality control.

Preparation of mitochondrial proteins for tryptic digest

Mitochondrial-enriched fractions were thawed on ice and diluted 1:1 with MIB containing 1x Protease Inhibitors Cocktail (Roche). LDS loading buffer (4x, Thermo) and Sample Reducing Agent (Thermo) were added at 1:4 and 1:10 ratios, respectively, and the samples were incubated at 95°C for 5 min and sonicated for 25 sec at 25% amplitude. The insoluble material was removed by centrifugation for 10 min at 16,000 x *g*, the supernatant was transferred to a fresh tube. The protein

concentration was determined by the BCA assay (Thermo), and adjusted to the same protein concentration in all samples with MIB containing LDS and sample reducing agent. Proteins (24 µg) per lane were resolved on 10% Novex Bis-Tris NuPAGE gel, which was then fixed for 30 min in 50% v/v MeOH, 40% v/v Acetic acid, stained with Coomassie R stain (0.25% w/v Coomassie R, 50% v/v MeOH, 40% v/v Acetic acid) for 20 min, and de-stained for 3 hours with 3-4 changes of the de-staining solution (5% v/v MeOH, 7.5% v/v Acetic acid), and then in deionised water for 2 hours, with gentle agitation at room temperature.

Organoids

Organoids were generated from isolated intestinal crypts as described (Carroll et al., 2017; Sato et al., 2009). Mice were euthanized, small intestine was collected, and flushed with ice-cold PBS. The small intestine was cut open, and the villi were removed with a coverslip. After washing with ice-cold PBS, the intestine was incubated with 3 mM EDTA in PBS for 20 min on a rocker in a cold room. Crypts were obtained by mechanical shaking, followed by centrifugation at 4°C for 3 min at 76 x *g* and 3 washes with PBS to remove single cells and microorganisms. The pellet was washed with cold Advanced DMEM/F12 (ADF) and resuspended in 3 ml pre-warmed TrypLE Express at 37°C for 5 min to break crypts into individual cells. Once crypts were broken up, 7 ml ADF+100 µl of Pen/Strep was added. The suspension was passed through a 40-µm cell strainer (BD Biosciences) to filter out any large aggregates of cells. Single cells were then collected by centrifugation at 1349 x *g* for 3 min. The pellet was resuspended in a small volume of the remaining supernatant, mixed with phenol-free Matrigel (BD Biosciences) and seeded into a 24-well plate. The plate was placed into a 37°C incubator to solidify the Matrigel, following which 0.5 ml of crypt medium was added to each well. The crypt medium had the following composition: advanced DMEM/F12 supplemented with 10 mM HEPES, 20 mM Glutamax-1, N2 supplement, B27 supplement, penicillin-streptomycin, TrypLE Express (all from Invitrogen), *N*-acetylcysteine (Sigma), growth factors (EGF, 50 ng/ml, Invitrogen; Noggin, 100 ng/ml, Peprotech), and *R*-Spondin conditioned medium (1:4). During the first 48 h, 3 µM Chiron99021 and 1 mM valproic acid (both from Sigma) and 10 µM Y27632 (Cambridge Bioscience) were also added. The

resulting organoid cultures were passaged by washing with cold ADF medium followed by mechanical breaking of the Matrigel and organoids using a pipette. After a further wash, the organoids were mixed with 100 µl of fresh Matrigel and grown on 24-well plates in 5% CO_2 at 37°C. For gene expression analysis, RNA was extracted from organoids growing in 3 individual wells; these organoids originated from one animal of each genotype.

To obtain mitochondria-enriched preparations from organoids for proteomic analysis, cultured organoids from 3 individual 7-10 week-old animals of each genotype, all on the C57BL/6 genetic background, were used. The organoids were harvested with Corning™ Cell Recovery Solution (Fisher Scientific) following the manufacturer's instructions. Mitochondria-enriched fractions were prepared from WT, N0 and KD C57/BL6 mice (n=3) as described (Frezza et al., 2007). Briefly, organoid pellets were reuspended with ice-cold mitochondria isolation buffer (10 mM Tris/MOPS, 1 mM EGTA/Tris, 200 mM sucrose, pH 7.4) and homogenized with glass-Teflon potter homogenizer on ice. The homogenate was subjected to centrifugation at 600 × *g* for 10 min at 4°C to remove nuclei. The supernatant was then subjected to centrifugation at 7,000 × *g* for 20 min at 4°C to pellet the mitochondrial fraction. The pellet was washed once with ice-cold mitochondria isolation buffer, mitochondria were resupended in the same buffer, and protein concentrations were determined by the BCA assay (Thermo). The resuspended mitochondria-enriched fractions were mixed with NuPAGE LDS Sample Buffer (Thermo) and heated at 70°C for 10 min. NuPAGE™ Sample Reducing Agent (Thermo) was added into the samples, and proteins (14 μg) were resolved on 10% SDS-PAGE gel (with NuPAGE – MOPS buffer, Thermo).

Cells

Primary mouse embryonic fibroblast (MEF) cells were prepared from wild-type, Nrf2-knockout, and Keap1-knockdown Skh-1 hairless mice (Knatko et al., 2015). MEF cells were cultured in plastic dishes coated for 30 min with 0.1% (w/v) gelatin before use and grown in Iscove’s modified Dulbecco's medium (with L-glutamine) (IMDM) supplemented with human recombinant EGF (10 ng/mL), 1 ×

insulin/transferring/selenium, and 10% (v/v) heat-inactivated fetal bovine serum (FBS, Thermo Scientific). Isogenic human colorectal cancer DLD1 cell lines with either Nrf2-knockout (Nrf2-KO) or Nrf2-gain-of-function (Nrf2-GoF) mutations and Nrf2-KO lung cancer A549 cells were generated using CRISPR/Cas9 genome editing as described (Torrente et al., 2017) and confirmed by sequencing. DLD1, A549 and human liver cancer HepG2 cells were grown in Dulbecco's Modified Eagle Medium (DMEM, Gibco, Thermo Scientific) that contains L-glutamine, sodium pyruvate, and high D-glucose content (4.5 g/L) supplemented with 10% (v/v) heat-inactivated FBS. The human colorectal cancer cell line Caco2 was cultured in DMEM supplemented with 10% (v/v) heat-inactivated FBS and 1% MEM Non-essential Amino Acid Solution (Sigma). The human normal lung fibroblast cell line IMR90 was grown in DMEM supplemented with 20% (v/v) heat-inactivated FBS and 2mM L-Glutamine (Gibco, Thermo Scientific). All cell cultures were maintained in 5% CO_2 at 37°C and were routinely tested to ensure that they were mycoplasma-free.

Small interfering RNA (siRNA) transfection

Caco2 and HepG2 cells were transfected with 20 nM ON-TARGET plus Smart Pool siRNA against human *NFE2L2* (L-003755-00-0005, Horizon Discovery) or ON-TARGET plus Non-targeting Control Pool (D-001810-10-50, Horizon Discovery) using Lipofectamine® RNAiMAX (Thermo Scientific) following manufacture’s instruction. In brief, siRNA targeting *NFE2L2*/non-targeting control and Lipofectamine® RNAiMAX were mixed in Opti-MEM (Gibco, Thermo Scientific) and incubated for 20 min at room temperature. At the same time, cells were trypsinized as normal and diluted to 1×10^5 cells per ml of medium. 500 μl of the RNAiMAX/siRNA/Opti-MEM was aliquoted into each well of a 6-well plate, to which 2 ml of the diluted cell suspension was added, and gently mixed. Cells were harvested at 2,3 and 4 days after transfection for further analysis.

Real-time quantitative PCR

Total RNA was extracted from cultured cells, organoids and mouse liver and colon using RNeasy Kit (Qiagen Ltd.). Omniscript RT Kit (Qiagen Ltd.) was then used to reverse-transcribe 500 ng of total RNA into cDNA. Real-time PCR was carried out on

Applied Biosystems QuantStudio™ 5 Real-Time PCR System. The TaqMan data for the mRNA species were normalized using mouse ribosomal protein lateral stalk subunit P0 (Rplp0), actin-beta, and 18S rRNA as internal controls. For human samples, human hypoxanthine phosphoribosyltransferase 1 (Hprt1) was used as an internal control. The TaqMan^{TM} Gene Expression Assay IDs (Thermo) used are listed below.

**Gene Name**	**Assay ID**
18S	Hs99999901_s1
Acaca (mouse)	Mm01304257_m1
Acadl (mouse)	Mm00599660_m1
Acadm (mouse)	Mm01323360_g1
Acads (mouse)	Mm00431617_m1
Acadvl (mouse)	Mm00444293_m1
Acly (mouse)	Mm01302282_m1
Acox2 (mouse)	Mm00446408_m1
Acss2 (mouse)	Mm00480101_m1
Actb (mouse)	Mm00607939_s1
Cd36 (mouse)	Mm00432403_m1
Ces1f (mouse)	Mm00523518_m1
Ces1g (mouse)	Mm00491334_m1
Cpt1a (mouse)	Mm01231183_m1
Elovl1 (mouse)	Mm01188316_g1
Elovl3 (mouse)	Mm00468164_m1
Elovl6 (mouse)	Mm00851223_s1
Fasn (mouse)	Mm00662319_m1
Gclc (mouse)	Mm00802655_m1
Gstp1 (mouse)	Mm04213618_gH
Nqo1 (mouse)	Mm01253561_m1
Rplp0 (mouse)	Mm00725448_s1
Scd1 (mouse)	Mm00772290_m1
Scd2 (mouse)	Mm01208542_m1
Slc25a20 (mouse)	Mm00451571_m1
ACOX2 (human)	Hs00185873_m1
AKR1B10 (human)	Hs00252524_m1
CES1 (human)	Hs00275607_m1
GCLC (human)	Hs00155249_m1
HPRT1 (human)	Hs02800695_m1
NFE2L2 (human)	Hs00975961_g1
NQO1 (human)	Hs00168547_m1

Immunoblotting

Frozen tissues (liver and colon) were pulverised under liquid nitrogen using a mortar and pestle. Colon tissue powder (15 mg) was homogenized in 10 volumes of ice-cold RIPA buffer (50 mM Tris-HCl, pH 7.5, 150 mM NaCl, 1% NP-40, 0.1% SDS, 1% sodium

deoxycholate), supplemented with EDTA-free protease inhibitors cocktail (Roche) on a rotator wheel for 1 hour at 4°C. Liver tissue powder (15 mg) was homogenized for 20 sec in 10 volumes of ice-cold assay buffer from the PicoProbe acetyl-CoA assay kit (Abcam, ab87546) supplemented with EDTA-free protease inhibitors cocktail (Roche) using rotor-stator homogeniser (Physcotron NS310-E3, Microtech, Japan). The insoluble material was removed by centrifugation for 10 min at 16,000 x *g* at 4°C. An aliquot of the supernatant was taken for determination of protein concentration by the bicinchoninic acid (BCA) assay (Thermo). To the remaining supernatant, 4 x LDS loading buffer (Thermo) was added to achieve a final 1 x concentration, and the protein concentration was adjusted using LDS in RIPA buffer to the same protein concentration in all samples. Sample Reducing Agent (Thermo) was added, and the samples were incubated at 70°C for 10 min prior to electrophoresis.

Cells were washed once with PBS before lysing in SDS Laemmli loading buffer (62.5 mM Tris-HCl, pH 6.8, 2% SDS, 10% glycerol, 0.02% Bromophenol Blue); the volume of lysis buffer was between 100-150 µl depending on the cell confluence. Lysates were then transferred into Eppendorf tubes, boiled for 5 min, sonicated for 20 sec at 20% amplitude using Vibra-Cell ultrasonic processor (Sonic). Protein concentrations were determined by the bicinchoninic acid (BCA) assay (Thermo). A solution of bromophenol blue (5%, v/v) was then added to each sample, and the volume was adjusted to achieve the same protein concentration in all samples. Proteins were resolved by electrophoresis using pre-cast 4-12% gradient NuPage™ gels (Life Technologies) or hand-cast, 8% Tris-Glycine gels, and transferred onto nitrocellulose membranes (Amersham Biosciences). Membranes were blocked in either 5% non-fat milk or 3% BSA for 45 min, on a rocker (60-70 rpm), at room temperature, and then incubated with the primary antibodies at 4°C on a rocker overnight.

Determination of autophagic flux

Parental A549 or Nrf2-KO A549 cells were seeded at a density of 3×10^5 cells per well of a 6-well plate. After 20-24 h, cells were treated with either vehicle (0.1% DMSO) or 10 nM Bafilomycin A (BAF) for 16 h. Following the treatment, the cells were washed thrice with PBS and lysed in 150 µL of SDS lysis buffer [50 mM Tris-HCl pH

6.8, 2% SDS (w/v), 10% Glycerol (v/v) and 0.005% Bromophenol Blue (w/v)]. The lysates were subjected to sonication for 20 sec at 20 % amplitude. Protein concentrations were determined using the BCA assay (Thermo), and equal amounts of protein (10-20 µg) from each sample was loaded into each well of a 15% Tris-Glycine SDS polyacrylamide gel and subjected to electrophoresis. Once the proteins were resolved on the gel, they were transferred onto 0.45-µm premium nitrocellulose membranes (Amersham Biosciences) using wet electroblotting transfer system (Bio-Rad). Subsequently, the membranes were blocked in PBST-milk [5% (w/v) non-fat milk dissolved in PBS-0.01 % Tween (v/v)] for 1 h at room temperature (RT). Following blocking, the membranes were incubated overnight at 4°C with either the LC3B or GAPDH antibody diluted in PBST-milk. Next, the membranes were washed thrice for 30 min with PBS-0.01% Tween and incubated with the respective fluorescently-labeled IRDye® secondary antibodies 1:20,000 (LI-COR) for 1 hour at room temperature, and were protected from light. After incubation with the secondary antibodies, the immunoblots were washed thrice for 30 min with PBS-0.01% Tween before scanning using the Odyssey CLx Near-Infrared Fluorescence Imaging System (LI-COR). The images obtained were analysed in the Image Studio software (Version 4.0.21).

Determination of triglycerides and acetyl-CoA

Triglycerides were determined using Trigyceride Assay Kit (Abcam, ab65336) according to the manufacturer's instructions for the colorimetric detection method. Briefly, frozen tissues were pulverised under liquid nitrogen followed by extraction in 5% NP-40 (10 µl per mg of tissue). Two µl of extract was used per assay well, in triplicates.

Acetyl-CoA was measured using PicoProbe Acetyl CoA Assay kit (Abcam, ab87546) according to the manufacturer's instructions for tissue samples. In brief, frozen tissues were pulverised under liquid nitrogen, homogenised in 1M ice-cold $HClO_4$ (2 µl per 1 mg tissue), the precipitants were removed by centrifugation for 10 min at 10,000 x *g* at 4°C, and the supernatants neutralized with 3M $KHCO_3$. 10 µl of clear supernatants were used per assay reaction.

Proteomics

For both proteomic experiments, proteins from mitochondrial preparations of three types, namely WT control (WT), Nrf2 knockout (Nrf2-KO), and Keap1 knockdown (Keap1-KD), were fractionated by SDS-PAGE and excised into two gel sections per lane. Peptides were extracted by tryptic digestion (Shevchenko et al., 2006), including alkylation with iodoacetamide. Peptide samples were analyzed by LC-MS/MS on a Q Exactive mass spectrometer (Thermo Scientific) coupled to an EASY-nLC 1000 liquid chromatography system via an EASY-Spray ion source (Thermo Scientific) running a 75 μm x 500 mm EASY-Spray column at 45°C. Data were acquired in the data-dependent mode. Full scan spectra (m/z 300–1800) were acquired with resolution R = 70,000 at m/z 200 (after accumulation to a target value of 1,000,000 with maximum injection time of 20 ms). The 10 most intense ions were fragmented by HCD and measured with a resolution of R = 17,500 at m/z 200 (target value of 500,000, maximum injection time of 60 ms) and intensity threshold of $2.1x10^4$. Peptide match was set to 'preferred' and a 40 second dynamic exclusion list was applied. For each experiment two elution gradients were used: Liver – 60 min and 240 min, Intestinal organoids – 140 min and 180 min.

Raw MS data files were processed using MaxQuant (v 1.6.1.0) with the built-in Andromeda peptide search engine (version 1.3.0.5) (Cox and Mann, 2008; Cox et al., 2011). The mouse uniprot protome was searched (downloaded October 2019 – 55153 entries). Enzyme specificity was set to trypsin-P. Cysteine carbamidomethylation was selected as a fixed modification with methionine oxidation and protein N-terminal acetylation as variable modifications. Initial maximum allowed mass deviation was set to 20 parts per million (ppm) for peptide masses and 0.5 Da for MS/MS peaks. The minimum peptide length was set to 7 amino acids and maximum size 4600 Da. A maximum of two missed cleavages were considered. A false discovery rate (FDR) of 1% was required at both the protein and peptide levels. Label-free quantification was selected and the 'match between runs' option was applied with a time window of two minutes.

The unfiltered proteinGroups.txt file (**Data S1**, Quantitative proteomics data) contained 6259 protein group entries, which after filtering for decoy proteins, those identified only by modified peptide(s), putative contaminants and those without a complete set of three LFQ values in at least one set of triplicates in at least one MS run, left 3752 proteins. Prior to further statistical analysis, LFQ values were further manually normalized within each MS run by median ratio of lane protein intensity/average of all lanes protein intensity for all common proteins for each slice (upper or lower). Once normalized, a single normalized LFQ value for each replicate in each MS run was calculated by the sum of the two slices normalized LFQ intensity for each peptide. This was necessary as output LFQ values were apparently not appropriately normalized when replicates were compared. Statistical analyses were performed using Perseus (v 1.6.1.1) (Tyanova et al., 2016). Data were separated into the four MS runs (two for liver and two for organoids samples). Zero values were replaced using Perseus default settings, and comparisons among cell types was performed using a two-tailed Student's t-test with cutoffs set at 10% FDR with an S0 value of 0.1. Data for all four MS runs were recombined into a single Excel file (**Data S1**, Quantitative proteomics data; "Accepted" sheet). The mass spectrometry proteomics data have been deposited to the ProteomeXchange Consortium via the PRIDE (Perez-Riverol et al., 2019) partner repository with the dataset identifier PXD021639.

STRING functional enrichment analyses were performed as follows. For both liver (1590 entries) and intestinal organoid (3335 entries) experiments, gene sets and quantitative data were uploaded to STRING (December 2019) using the 'Proteins with values/ranks" tool (Szklarczyk et al., 2019). This looks for functional enrichments among proteins deviating away from $\log_2$ ratio=0, and therefore cellular functions potentially regulated by the experimental conditions (see **Data S2**, STRING functional group enrichment analysis for a full list of enrichments). Networks of proteins with high enrichment scores (extreme ratios), and low FDR values (high statistical significance) were selected for presentation.

Metabolomics

Metabolites were extracted using the methanol/chloroform/water (2:2:1; v/v) method described previously (Wang et al., 2015; West et al., 2016). Briefly, 50 mg of wet weight tissue was mixed with 600 µl of $CH_3OH/CHCl_3$ (2:1; v/v), and the samples were homogenized with a Tissuelyser (Qiagen, UK) for 5 min at a frequency of 20/s and sonicated for 15 min. Water and chloroform (each of 200 µl) were added to the samples before centrifugation at 13,300 rpm for 20 min. The resulting aqueous and organic phases were separated from the protein pellets. The extraction procedure was repeated on the remaining protein pellets. Both organic and aqueous phases were collected and evaporated to dryness. The dried samples were stored at −80°C until further analysis.

FAME Analysis

50 µl of D-25 tridecanoic acid (200 µM in chloroform), 650 µl of chloroform/methanol (1:1 v/v) and 125 µl BF_3/methanol (Sigma-Aldrich) was added to 100 µl organic extract dissolved in chloroform/methanol (1:1 v/v) (a quarter of the organic material extracted for each sample). The samples were then incubated at 80 ºC for 90 min. 500 µl H_2O and 1 ml hexane were added and each vial mixed. The organic layer was evaporated to dryness before reconstitution in 200 µl hexane for analysis. Using a Trace GC Ultra coupled to a Trace DSQ II mass spectrometer (Thermo Scientific, Hemel Hempstead, UK), 4 µl of the derivatised organic metabolites were injected onto a TR-fatty acid methyl ester (FAME) stationary phase column (Thermo Electron; 30 m × 0.25 mm ID × 0.25 µm; 70% cyanopropyl polysilphenylene-siloxane) with a split ratio of 20. The injector temperature was 230°C and the helium carrier gas flow rate was 1.2 ml/min. The column temperature was 60°C for 2 min, increased by 15°C/min to 150°C, and then increased at a rate of 4°C/min to 230°C (transfer line = 240°C; ion source = 250°C, EI = 70 eV). The detector was turned on after 240 s, and full-scan spectra were collected using 3 scans/s over a range of 50–650 *m*/*z*. Peaks were assigned using the Food Industry FAME Mix (Restek 6098).

LC-MS analysis of aqueous metabolites

Half of the extracted aqueous samples were reconstituted in 7:3 acetonitrile: 0.1 M aqueous ammonium carbonate containing 2 µM [$^{13}C_{10}{}^{15}N_5$] adenosine monophosphate, [$^{13}C_{10}{}^{15}N_5$] adenosine triphosphate, 10 µM [$^{13}C_4$] succinic acid and 10 µM [$^{13}C_5{}^{15}N_5$] glutamic acid (all from Sigma Aldrich except the glutamic acid from Cambridge Isotope Laboratories) as internal standards. The samples were vortexed then sonicated for 15 min followed by centrifugation at 21,000 x *g* to pellet any remaining undissolved material. They were analyzed on a Quantiva triple stage quadrupole mass spectrometer coupled to a Vanquish Horizon (all analytical instrument combinations supplied by Thermo Fisher Scientific), using a bridged ethylene hybrid (BEH) amide hydrophilic interaction liquid chromatography (HILIC) column, as previously described (Cader et al., 2020). The strong mobile phase (A) was 100 mM ammonium carbonate, the weak mobile phase was acetonitrile (B) with water:acetonitrile (1:1) being used for the needle wash. The LC column used was the BEH amide column (150 × 2.1 mm, 1.7 µm, Waters). The following linear gradient was used: 20% A in acetonitrile for 1.5 min followed by an increase to 60% A over 2.5 min with a further 1 min at 60% A after which the column was re-equilibrated for 1.9 min. After each chromatographic run the column was washed with 30 column volumes of water:acetonitrile (6:4) followed by a further 10 column volumes of acetonitrile:water (95:5) for storage. The total run time was 7 min, the flow rate was 0.6 mL/min and the injection volume was 5 µL. In order to resolve pentose phosphates for the identification of ribose-1-phosphate a shallower gradient was employed: 30% A in acetonitrile for 2.0 minutes followed by an increase to 50% A over 3.0 minutes with re-equilibration for 1.9 minutes.

GC-MS and LC-MS data processing

GC–MS and LC-MS chromatograms were analysed using Xcalibur, version 2.0 (Thermo Fisher), integrating each peak individually. GC-MS Peaks were normalised to total area, while LC-MS peaks were normalised to the internal standard.

Multivariate analysis of metabolite profiles

The set of metabolic profiles obtained were analysed by multivariate analysis. Datasets were imported into SIMCA-P 15.0 (Sartorius AG, Gottingen, Germany) for processing using PCA and PLS-DA (a regression extension of PCA used for supervised classification). GC-MS data were scaled to unit variance by dividing each variable by $1/(S_k)$.

Univariate statistical analysis

Univariate statistical analyses were performed using Excel (Microsoft). Values are expressed as mean ± S.D. and the significance level was set at $p<0.05$. For comparisons of three groups, one-way ANOVA was used with a Tukey test with Bonferroni correction as a post-test.* 0.05> p < 0.01; ** 0.01> p < 0.001.

Supplemental References

Cader, M.Z., de Almeida Rodrigues, R.P., West, J.A., Sewell, G.W., Md-Ibrahim, M.N., Reikine, S., Sirago, G., Unger, L.W., Iglesias-Romero, A.B., Ramshorn, K., et al. (2020). FAMIN Is a Multifunctional Purine Enzyme Enabling the Purine Nucleotide Cycle. Cell *180*, 278-295 e223.

Carroll, T.D., Langlands, A.J., Osborne, J.M., Newton, I.P., Appleton, P.L., and Nathke, I. (2017). Interkinetic nuclear migration and basal tethering facilitates post-mitotic daughter separation in intestinal organoids. J Cell Sci *130*, 3862-3877.

Cox, J., and Mann, M. (2008). MaxQuant enables high peptide identification rates, individualized p.p.b.-range mass accuracies and proteome-wide protein quantification. Nat Biotechnol *26*, 1367-1372.

Cox, J., Neuhauser, N., Michalski, A., Scheltema, R.A., Olsen, J.V., and Mann, M. (2011). Andromeda: a peptide search engine integrated into the MaxQuant environment. J Proteome Res *10*, 1794-1805.

Frezza, C., Cipolat, S., and Scorrano, L. (2007). Organelle isolation: functional mitochondria from mouse liver, muscle and cultured fibroblasts. Nat Protoc *2*, 287-295.

Honda, T., Yoshizawa, H., Sundararajan, C., David, E., Lajoie, M.J., Favaloro, F.G., Jr., Janosik, T., Su, X., Honda, Y., Roebuck, B.D., et al. (2011). Tricyclic compounds containing nonenolizable cyano enones. A novel class of highly potent anti-inflammatory and cytoprotective agents. J Med Chem *54*, 1762-1778.

Knatko, E.V., Ibbotson, S.H., Zhang, Y., Higgins, M., Fahey, J.W., Talalay, P., Dawe, R.S., Ferguson, J., Huang, J.T., Clarke, R., et al. (2015). Nrf2 Activation Protects

against Solar-Simulated Ultraviolet Radiation in Mice and Humans. Cancer Prev Res (Phila) *8*, 475-486.

Perez-Riverol, Y., Csordas, A., Bai, J.W., Bernal-Llinares, M., Hewapathirana, S., Kundu, D.J., Inuganti, A., Griss, J., Mayer, G., Eisenacher, M., et al. (2019). The PRIDE database and related tools and resources in 2019: improving support for quantification data. Nucleic Acids Res *47*, D442-D450.

Sato, T., Vries, R.G., Snippert, H.J., van de Wetering, M., Barker, N., Stange, D.E., van Es, J.H., Abo, A., Kujala, P., Peters, P.J., et al. (2009). Single Lgr5 stem cells build crypt-villus structures in vitro without a mesenchymal niche. Nature *459*, 262-265.

Shevchenko, A., Tomas, H., Havlis, J., Olsen, J.V., and Mann, M. (2006). In-gel digestion for mass spectrometric characterization of proteins and proteomes. Nat Protoc *1*, 2856-2860.

Szklarczyk, D., Gable, A.L., Lyon, D., Junge, A., Wyder, S., Huerta-Cepas, J., Simonovic, M., Doncheva, N.T., Morris, J.H., Bork, P., et al. (2019). STRING v11: protein-protein association networks with increased coverage, supporting functional discovery in genome-wide experimental datasets. Nucleic Acids Res *47*, D607-D613.

Taguchi, K., Maher, J.M., Suzuki, T., Kawatani, Y., Motohashi, H., and Yamamoto, M. (2010). Genetic analysis of cytoprotective functions supported by graded expression of Keap1. Mol Cell Biol *30*, 3016-3026.

Torrente, L., Sanchez, C., Moreno, R., Chowdhry, S., Cabello, P., Isono, K., Koseki, H., Honda, T., Hayes, J.D., Dinkova-Kostova, A.T., et al. (2017). Crosstalk between NRF2 and HIPK2 shapes cytoprotective responses. Oncogene *36*, 6204-6212.

Tyanova, S., Temu, T., Sinitcyn, P., Carlson, A., Hein, M.Y., Geiger, T., Mann, M., and Cox, J. (2016). The Perseus computational platform for comprehensive analysis of (prote)omics data. Nature methods *13*, 731-740.

Wang, X., West, J.A., Murray, A.J., and Griffin, J.L. (2015). Comprehensive metabolic profiling of age-related mitochondrial dysfunction in the high-fat-fed ob/ob mouse heart. J Proteome Res *14*, 2849-2862.

West, J.A., Beqqali, A., Ament, Z., Elliott, P., Pinto, Y.M., Arbustini, E., and Griffin, J.L. (2016). A targeted metabolomics assay for cardiac metabolism and demonstration using a mouse model of dilated cardiomyopathy. Metabolomics *12*, 59.
